# A genome sequence resource for the genus *Passiflora*, the genome of the wild diploid species *Passiflora organensis*

**DOI:** 10.1002/tpg2.20117

**Published:** 2021-07-23

**Authors:** Zirlane Portugal Costa, Alessandro Mello Varani, Luiz Augusto Cauz‐Santos, Mariela Analía Sader, Helena Augusto Giopatto, Bruna Zirpoli, Caroline Callot, Stephane Cauet, Willian Marande, Jessica Luana Souza Cardoso, Daniel Guariz Pinheiro, João Paulo Kitajima, Marcelo Carnier Dornelas, Andrea Pedrosa Harand, Helene Berges, Claudia Barros Monteiro‐Vitorello, Maria Lucia Carneiro Vieira

**Affiliations:** ^1^ Dep. de Genética, Escola Superior de Agricultura “Luiz de Queiroz” Univ. de São Paulo Piracicaba 13418–900 Brazil; ^2^ Dep. de Tecnologia, Faculdade de Ciências Agrárias e Veterinárias Univ. Estadual Paulista Jaboticabal 14884–900 Brazil; ^3^ Dep. de Botânica Univ. Federal de Pernambuco Recife 50670–901 Brazil; ^4^ Dep. de Biologia Vegetal, Instituto de Biologia Univ. Estadual de Campinas Campinas 13083–862 Brazil; ^5^ Institut National de la Recherche Agronomique Centre National de Ressources Génomique Végétales Castanet‐Tolosan 31326 France; ^6^ Mendelics Análise Genômica AS São Paulo 04013–000 Brazil; ^7^ Present address: Dep. of Botany and Biodiversity Research Univ. of Vienna Vienna 1030 Austria

## Abstract

The genus *Passiflora* comprises a large group of plants popularly known as passionfruit, much appreciated for their exotic flowers and edible fruits. The species (∼500) are morphologically variable (e.g., growth habit, size, and color of flowers) and are adapted to distinct tropical ecosystems. In this study, we generated the genome of the wild diploid species *Passiflora organensis* Gardner by adopting a hybrid assembly approach. *Passiflora organensis* has a small genome of 259 Mbp and a heterozygosity rate of 81%, consistent with its reproductive system. Most of the genome sequences could be integrated into its chromosomes with cytogenomic markers (satellite DNA) as references. The repeated sequences accounted for 58.55% of the total DNA analyzed, and the *Tekay* lineage was the prevalent retrotransposon. In total, 25,327 coding genes were predicted. *Passiflora organensis* retains 5,609 singletons and 15,671 gene families. We focused on the genes potentially involved in the locus determining self‐incompatibility and the MADS‐box gene family, allowing us to infer expansions and contractions within specific subfamilies. Finally, we recovered the organellar DNA. Structural rearrangements and two mitoviruses, besides relics of other mobile elements, were found in the chloroplast and mt‐DNA molecules, respectively. This study presents the first draft genome assembly of a wild *Passiflora* species, providing a valuable sequence resource for genomic and evolutionary studies on the genus, and support for breeding cropped passionfruit species.

## Core Ideas

- *Passiflora organensis* has a small genome of 259 Mbp.
- We estimate that the repetitive content of the *P. organensis* genome is approximately 59%.
- We predicted 25,327 protein‐coding genes and genes involved in self‐incompatibility.
- Organelle DNAs were recovered; two mitoviruses and relics of mobile elements occurred in mtDNA.
- This study provides a promising resource for comparative genomic analysis of *Passiflora*.

**Abbreviations**
ANI: average nucleotide identity
CDS: coding regions
FISH: fluorescence in situ hybridization
GO: Gene Ontology
K: synonymous nucleotide substitution rate
LTR: long terminal repeat
LTR‐RT: long terminal repeat–retrotransposon
MIKC: MADS intervening keratin‐like and *C*‐terminal
mya: million years ago
NADH: nicotinamide adenine dinucleotide hydrogen
SI: self‐incompatibility
SLG: S‐locus glycoproteins
SRK: S‐locus receptor kinases
TE: transposable element
WGD: whole‐genome duplication.

## 1 INTRODUCTION

*Passiflora* (Passifloraceae, Malpighiales) is a neotropical genus encompassing hundreds of species with widespread distribution in the American continent, including the Amazonian and Andean regions. Population pressure in all these regions is high, raising considerable concern over pollinator decline (e.g., bees, bats, and hummingbirds) and conservation of *Passiflora* diversity. There are four main subgenera: *Astrophea* (57 species), *Decaloba* (220 species), *Deidamioides* (13 species), and the *Passiflora* subgenus (240 species), which contains several self‐incompatible species (Ulmer & MacDougal, 2004). Although some 50 species are traditionally cropped in temperate regions (e.g., the United States), most are grown in tropical climates to produce fruit (Cerqueira‐Silva et al., 2015; Zerbini et al., 2008).

*Passiflora* species morphology is highly variable, with a 10‐fold variation in genome size (1C DNA content = 0.212 pg in *Passiflora organensis* Gardner up to 2.68 pg in *Passiflora quadrangularis* L. (Souza et al., 2004; Yotoko et al., 2011) that is not attributable to polyploidy. Although it is the species with the smallest genome species, *P. organensis* (subgenus *Decaloba*) seems to be evolving by diversification of different repeat types; the largest, *P. quadrangularis* (subgenus *Passiflora*), evolved by accumulating retrotransposons, especially *Angela* and *Tekay* elements, which comprise most of its genome (Sader et al., 2021).

The publication of several draft plant genomes has undoubtedly provided valuable resources for the scientific communities conducting top‐level research in their respective fields. For instance, advanced sequencing technologies have resulted in near‐complete, high‐quality chromosome‐scale genome assemblies at minimal cost (Pham et al., 2020). However, we are only just beginning to investigate the genomic information on underutilized crops (e.g., Gioppato et al., 2019).

Against this backdrop, a genomic library was built covering around six times the genome length of the sour passionfruit (*Passiflora edulis* Sims) (Santos et al., 2014), whose nuclear genome size was estimated at 1.258 pg (expressed as 1C) or 1,232 Mbp (Yotoko et al., 2011). On the basis of gene richness, we selected and completely sequenced over 100 inserts. Sequence data were assembled from long sequence reads, and structural sequence annotation resulted in the prediction of some 1,900 genes (Munhoz et al., 2018). The dataset was also subjected to transposable element (TE) discovery and characterization of long terminal repeat (LTR)–retrotransposon (LTR‐RT) evolutionary lineages. Most of the TEs were located in intergenic spaces (∼70%), although some overlapped genes. Long terminal repeat retroelements predominated, consisting mainly of *Gypsy* elements, with over‐representation of the *RLG_peDel* (or *Tekay* lineage according to Neumann et al. [2019]), and single elements of *P. edulis* were obtained for the first time (Costa et al., 2019). Additionally, Sader, Dias, et al. (2019), who used a *Gypsy* element sequence as a probe for in situ hybridization, found a dispersed and uniform distribution pattern along *P. edulis* chromosomes, implying that they were abundant and could have significantly influenced the genome size. Other information on the nuclear genome of *P. edulis* was derived from low‐coverage sequencing data, facilitating microsatellite marker development (Araya et al., 2017) and providing cytogenomic markers, especially satellite sequences that are possibly associated with 5S and 35S rDNA or subtelomeres (Pamponét et al., 2019).

Very recently, a ∼1,341.7‐Mbp chromosome‐scale genome assembly of the purple passionfruit (*P. edulis*) was reported (Xia et al., 2021), with 98.91% of the assembly assigned to nine pseudochromosomes. The genome harbors 23,171 protein‐coding genes, and most of the assembled sequences are repetitive, with a predominance of LTRs, confirming our previous findings. Moreover, important gene families were identified by integrating transcriptomic and metabolomic analyses, centered on genes involved in the synthesis of volatile organic compounds and providing insights for improving the flavor of fresh fruit.

**FIGURE 1:**
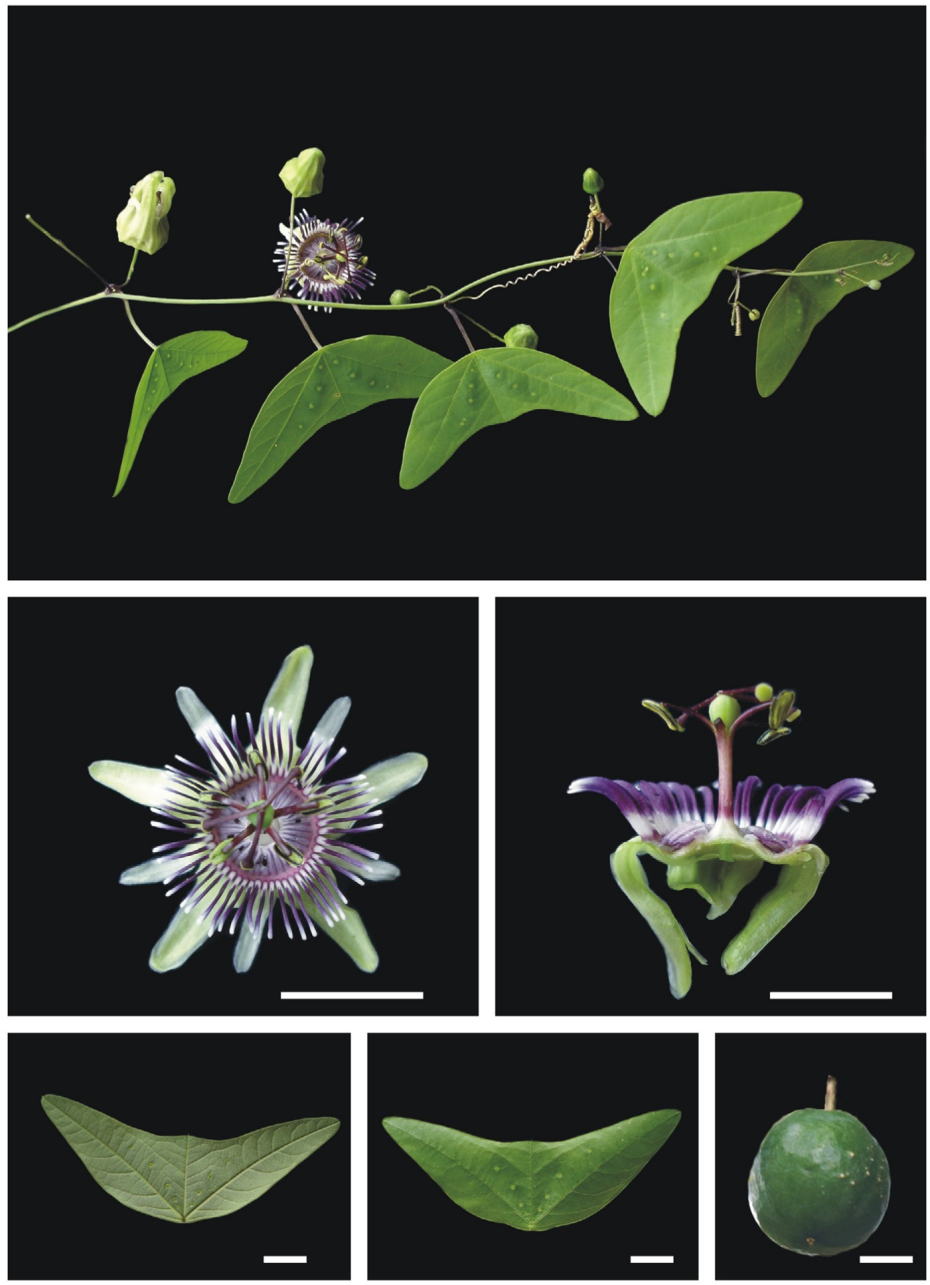
Macromorphology of *Passiflora organensis*. The habit is shown in the upper panel. The middle images show the top (left) and lateral views of a longitudinal section (right) of a flower at anthesis. The lower panels show a leaf from the abaxial (left) and adaxial views (middle), and a fruit from the lateral view (right). Bars: 1.5 cm

This interesting scenario inspired us to sequence the whole genome of the wild diploid species *P. organensis*, which has the smallest known *Passiflora* genome, but whose overall morphology is very representative of the genus (Figure 1). A draft genome assembly was generated on the basis of a combination of the genomic and transcriptomic data associated with cytogenomic markers. The availability of a reference sequence will greatly facilitate further progress in comparative genomics and molecular genetics, geared towards assisting passion fruit breeding initiatives. It will also serve as a valuable tool for evolutionary analyses of *Passiflora*, providing a scaffold for genome assembly, variant calling, RNA read alignment, chromosome molecular mapping, gene annotation, and functional analysis, especially for comparison with the purple passionfruit genome (Xia et al., 2021).

*Passiflora organensis* and the cultivated species *P. edulis* belong to the same genus but different subgenera (*Decaloba* and *Passiflora*, respectively). As expected, they share some common physiological and phenological characteristics (both are perennial lianas and flower in response to longer days), but diverge in others. *Passiflora organensis* produces small fruits with almost no juice, whereas *P. edulis* produces large fruits with juice‐rich arillate seeds. In this respect, the availability of both genomes will further our understanding of important domesticated fruit traits and the underlying mechanisms.

Finally, it is well known that some *Passiflora* species have a self‐incompatibility system controlled by sporophytic and gametophytic mechanisms, and/or have coevolved with butterflies of the subfamily Heliconiinae. The occurrence of structures mimicking heliconiine eggs, the production of extrafloral nectar to attract the predators of *Passiflora* herbivores, and the wide diversity of defense compounds, including cyanogenic glucosides [reviewed in Krist (2020)], are strong arguments in favor of generating an additional reference genome to facilitate functional studies.

## 2 MATERIAL AND METHODS

A single *P. organensis* plant was used for DNA isolation. It was field‐collected in the Biological Reserve of Alto da Serra de Paranapiacaba (Santo André, São Paulo; 23.77309 S, 46.3017 W). This municipality is located in the Atlantic forest biome of Southeastern Brazil. The plant was kept in a greenhouse at our laboratory (Piracicaba, São Paulo; 22.71027 S, 47.63286 W). Its accession number (AF82F07) is registered in the Sistema Nacional de Gestão do Patrimônio Genético e do Conhecimento Tradicional Associado, Brazil.

### 2.1 Cytogenomics

Root tips obtained from potted plants were pretreated with 2 mM 8‐hydroxyquinoline for 4.5 h at 10 °C, fixed in ethanol–acetic acid (3:1 v/v), and stored in a fixative at –20 °C. The root tips were then digested in a solution containing 2% cellulase and 20% pectinase (w/v) for 90 min at 37 °C, and chromosome preparations were performed according to Carvalho and Saraiva (1993). Slides were selected after staining with 2 μg ml^–1^ 4′,6‐diamidino‐2‐phenylindole (Sigma) in 50% glycerol (Cabral et al., 2006).

**Core Ideas**

- *Passiflora organensis* has a small genome of 259 Mbp.
- We estimate that the repetitive content of the *P. organensis* genome is approximately 59%.
- We predicted 25,327 protein‐coding genes and genes involved in self‐incompatibility.
- Organelle DNAs were recovered; two mitoviruses and relics of mobile elements occurred in mtDNA.
- This study provides a promising resource for comparative genomic analysis of *Passiflora*.

Four satDNA probes were used for fluorescent in situ hybridization (FISH) experiments (Sader et al., 2021) (PorSat01‐161, PorSat04‐1800, PorSat07‐1004, and PorSat09‐1200) and labeled with 5‐amino‐propargyl‐2′‐deoxyuridine 5′‐triphosphate and cyanine red fluorescent dye (GE Healthcare). The FISH procedure applied to mitotic chromosomes was essentially as described in Fonsêca et al. (2010). The hybridization mix consisted of 50% (v/v) formamide, 10% (w/v) dextran sulfate, 2× SSC, and 2 to 5 ng μl^–1^ of the probes. The slides were denatured for 5 min at 75 °C and hybridized for 24 h at 37 °C. The final stringency was 76%. The slides were mounted with Vectashield (Vector) containing 2 μg ml^–1^ 4′,6‐diamidino‐2‐phenylindole. Images were captured on an epifluorescence microscope (Leica Microsystems) running Leica QFISH software and a DMLB microscope running Leica Las‐AF software. For final processing, the images were uniformly adjusted for brightness and contrast only, using Adobe Photoshop version 10.0. Three complete metaphase cells were used to measure the short and long arms of the individual chromosomes identified by satDNA probes with the Adobe Photoshop measurement tool. Chromosomes were identified and classified according to their length and arm ratio, as described in Guerra (1986).

### 2.2 DNA isolation, library construction, and sequencing of *P. organensis* DNA

Genomic DNA was extracted from young leaves to obtain high‐quality DNA for long‐ and short‐read sequencing. For PacBio long‐read sequencing, high molecular weight DNA was extracted via a modified version of the cetyltrimethylammonium bromide method adapted from Murray and Thompson (1980) and Carlson's lysis buffer (Carlson et al., 1991), followed by DNA purification via the Qiagen Genomic Tip 500/G protocol (Qiagen, Germany) following the manufacturer's instructions. DNA quality and quantity were checked in Nanodrop and Qubit (Thermo Fisher Scientific), and DNA fragment sizes were analyzed by pulsed‐field electrophoresis and in a Fragment Analyzer (Agilent Technologies).

The PacBio library was constructed using 5 g of DNA, and final sizing was performed to select fragments ranging from 20 to 150 kb. The DNA was then sequenced in eight runs in a single‐molecule real‐time cell and via P6‐C4 chemistry on the PacBio RSII (Pacific Biosciences) at the Uppsala NGI Platform (Uppsala University, Sweden). Finally, the raw data were filtered to obtain high‐quality reads (reads with a quality of <0.75 and a length of <500 bp were discarded). For Illumina short‐read sequencing, DNA was extracted by the cetyltrimethylammonium bromide method adapted from Murray and Thompson (1980) and used to construct three PCR‐free libraries with the Illumina TruSeq kit. Sequencing was run on an Illumina HiSeq platform with the Rapid Run Version 2 (Illumina) protocol to yield 250‐bp paired end reads. The Illumina raw reads were trimmed with Trimmomatic software (Bolger et al., 2014), discarding all reads with a PHRED value (a measure for base quality in DNA sequencing) lower than 24.

We also performed one run of Nanopore sequencing to complete the assembly of the mitochondrial genome. MinION libraries were prepared with the Oxford Nanopore 1D Genomic DNA Ligation kit (SQKLSK109) (Oxford Nanopore Technologies) and MinION protocol (version IDI_S1006_v1_revB_18Apr2016), followed by sequencing on a Nanopore MinION platform (MKE_1013_v1_revAR_11Apr2016).

### 2.3 Optical map construction

For BioNano optical genome mapping, ultra‐high molecular weight DNA was extracted at the Centre National de Ressources Génomiques Végétales (Toulouse, France), according to the Bionano IrysPrep Reagent Kit protocol (Bionano Genomics). Briefly, fresh leaves (∼0.5 g) were used for nucleus isolation and gradient density purification. High molecular weight DNA was then extracted from an agarose plug containing the nuclei isolated and subjected to Proteinase K digestion, followed by RNA digestion. Finally, the DNA was recovered, dialyzed, and then quantified in Qubit (Thermo Fisher Scientific). The DNA was loaded into an Irys chip (Bionano Genomics) and optically mapped in a BioNano Genomics Irys system according to the manufacturer's instructions.

### 2.4 RNA extraction for *P. organensis* transcriptome sequencing

Tissues from young leaves, stems, and floral buds were collected and immediately placed in liquid nitrogen. RNA was extracted via the Trizol protocol (Invitrogen) according to the manufacturer's instructions. The quality of the RNA was assessed on the basis of the RNA integrity numbers, computed on a Bioanalyzer 2100 (Agilent Technologies) and also by 1.2% agarose gel electrophoresis. This RNA provided a basis for constructing three libraries, one for each tissue (leaf, stem, and bud), with the Illumina TruSeq Stranded mRNA kit. Libraries were pooled and sequenced at 2× 100 bp in a half lane on the Illumina HiSeq2500 platform at the Center for Functional Genomics of the University of São Paulo, Piracicaba, Brazil.

### 2.5 Genome assembly

The genome was assembled in five steps (Supplemental Figure S1). In Step 1, FALCON‐Unzip Version 2.2.4 (Chin et al., 2016) was used to assemble the reads of eight PacBio Sequel single‐molecule real‐time cells (2,502,979 reads). In Step 2, SPACE‐LongRead Version 1.1 (Boetzer & Pirovano, 2014) corrected the PacBio reads (489,056) and expanded the assembled scaffolds. In Step 3, the trimmed 2× 250‐bp Illumina reads (89,918,970) were used to polish and correct the genome assembly with POLCA Version 3.4.2 (Zimin & Salzberg, 2020). In Step 4, RefAligner (Bionano Genomics) and runBNG Version 2.0 (Yuan et al., 2017) assembled the BioNano optical maps with the previously polished assembly reference. In the final step (Step 5), runBNG used the assembled optical map and polished the assembly for input to the Hybrid Scaffold pipeline (Bionano Genomics). In addition, taking *Populus trichocarpa* Torr. & A.Gray ex Hook. and *Manihot esculenta* Crantz. as references (Supplemental Table S1). RaGOO (Alonge et al., 2019) was used for performing additional reference‐guided scaffolding steps (parameters: a minimum unique alignment length of 50,000 bp and a maximum inferred gap size of 10,000 bp). Assembly gaps were closed with GapFiller Version 2.1.2 (Nadalin et al., 2012) and LR_GapCloser Version 1.1 (Xu et al., 2018), for the trimmed Illumina and PacBio reads, respectively. Benchmarking Universal Single‐Copy Orthologs Version 4.0.2 (Seppey et al., 2019) and the Embryophyta odb10 database were used to assess genome completeness. Genome assembly quality was also assessed on the basis of the LTR Assembly Index, computed according to Ou et al. (2018).

Repetitive sequences were investigated via the dot‐blot method to visualize two sequences and identify closely similar regions after sequence alignment. Default parameters were used for pairwise alignment of the scaffolds run on MUMmer4 (Marçais et al., 2018). This approach allowed us to map the centromeric regions.

GenomeScope Version 1.0.0 (Vurture et al., 2017) and Smudgeplot Version 0.2.4 (Ranallo‐Benavidez et al., 2020) were used to estimate the coverage, ploidy, and heterozygosity of the *P. organensis* genome. The diploid model was used to fit the results and default parameters for analyzing data on 89,918,970 Illumina reads.

### 2.6 Transcriptome assembly

*P. organensis* and *P. edulis* transcriptomes generated by the RNAseq approach were assembled with the Trinity tool Version 2.11.0 (Grabherr et al., 2011) with the default parameters. Coding regions within transcripts were identified by the TransDecoder pipeline Version 5.5.0 (https://github.com/TransDecoder/TransDecoder).

### 2.7 Transposable element discovery

De novo detection of TEs was run in REPET Version 3.0 (Jouffroy et al., 2016; Quesneville et al., 2005), and the bundled PASTEC tool Version 3.0 (Hoede et al., 2014) used to classify TEs at the order level on the basis of the Wicker hierarchical system (Wicker et al., 2007). The elements identified by REPET were manually curated and characterized into superfamilies and evolutionary lineages with DANTE (Domain‐based ANnotation of Transposable Elements) Version 1.0.1, available on the RepeatExplorer server (Novák et al., 2013; https://repeatexplorer‐elixir.cerit‐sc.cz/), and the Viridiplantae database Version 3.0 (Neumann et al., 2019). Transposable element masking was performed by RepeatMasker Version 4.0.7 (http://www.repeatmasker.org) with the “‐s ‐cutoff 260” parameter. The TEannot pipeline in the REPET package was also used to generate TE annotations in GFF3 file format.

To estimate when the LTR‐RT lineages were inserted into the *P. organensis* genome, sequences were used as input to LTR_FINDER Version 1.0.7 (Xu & Wang, 2007) to search for full‐length elements and compute the divergence (K) between LTRs. Insertion time (*t*) was then calculated for each full‐length element according to the formula: *t* = *K*/2*r* (Yin et al., 2015), where *t* is the insertion time in million years ago (mya), *K* is the number of nucleotide substitutions per site, and *r* is the nucleotide substitution rate. A value of 1.5 × 10^−8^ was assigned to *r*, as reported for the *Arabidopsis thaliana* (L.) Heyhn. chalcone synthase and alcohol dehydrogenase genes (Koch et al., 2000) used for dating the LTR‐RTs in *P. edulis* (Costa et al., 2019). In order to investigate the incorporation of entire genes or fragments thereof into TEs, the sequences were scanned for conserved domains in the Conserved Domain Database Version 3.18 (Marchler‐Bauer et al., 2017) public resource using the Conserved Domain Database search interface (https://www.ncbi.nlm.nih.gov/Structure/cdd/wrpsb.cgi), with default parameters.

### 2.8 Genome annotation

For gene prediction, the BRAKER Version 2 pipeline (Hoff et al., 2016) was used for *P. organensis*, inputting the transcriptome and protein data of very closely related species derived from the Benchmarking Universal Single‐Copy Orthologs searches and OrthoDB (Kriventseva et al., 2019) (–etpmode and –softmasking modes). The PASA pipeline Version 2.4.1 (Haas et al., 2003) produced spliced alignment assemblies based on the RNA‐seq data. EVidence Modeler Version 1.1.1 (Haas et al., 2008) combined with BRAKER2 Version 2.1.5 outputs (augustus.ab.initio, augustus.hints, and braker), and the transcript alignments were then used. Finally, two PASA pipeline iterations updated the EVidence Modeler consensus predictions, adding untranslated region annotations and models for the alternatively spliced isoforms. Functional annotation was performed with OmicsBox Version 1.4.10 (https://www.biobam.com/omicsbox/), InterProScan Version 5.46‐81.0 (Jones et al., 2014), and EggNOG‐mapper Version 1.0.3 (Huerta‐Cepas et al., 2017) with the EggNOG database Version 5.0 (Huerta‐Cepas et al., 2019). The UniProtKB/TrEMBL (The UniProt Consortium, 2017) Viridiplantae database was used for protein function assignment.

Gene ontology (GO) term enrichment analysis across different sets of genes was run with the MCScanX algorithm (Wang et al., 2012), which classifies genes as singletons and dispersed, proximal, tandem, and segmental or whole‐genome duplication (WGD) duplicates according to the copy number and genomic distribution. The following criteria were applied: (a) singletons were single‐copy genes, (b) adjacent genes were tandem, (c) genes separated by 10 or fewer genes were proximal, (d) genes separated by more than 10 genes were dispersed, and (e) the anchor genes in collinear blocks were WGD genes. We then identified over‐represented GO terms in these gene sets with GOATOOLS software (Klopfenstein et al., 2018), with a corrected *p* value of <.01 as the threshold for determining significant overrepresentation.

In addition, orthogroups were defined to assess common protein‐coding genes, and all gene sequences of Malpighiales species (*P. organensis*, *P. trichocarpa*, *Salix purpurea* L., *Ricinus communis* L., and *M. esculenta*) were used as input to OrthoVenn2 (Xu et al., 2019), set to the default parameters. To identify the genes potentially involved in self‐incompatibility, BlastP searches using Brassicaceae reference sequences of the S‐locus and partial *P. edulis* sequences (Madureira et al., 2014) were run to recognize homologs in the predicted *P. organensis* protein dataset. Sequences were identified on the basis of similarity and a domain structure resembling S‐locus glycoproteins (SLG) and S‐locus receptor kinases (SRK). The linear arrangements of domains (protein architecture) corroborated Xing et al. (2013) and were identified in the NCBI Conserved Domain Database (Lu et al., 2020). Variant domain architectures were also noted.

### 2.9 Annotation of noncoding RNA

All noncoding RNA classes were scanned with a query set from the Rfam database and cmsearch software in Infernal toolkit Version 1.1.2 (Nawrocki & Eddy, 2013). The following tools were used for class‐specific annotation of ncRNAs: (a) tRNAscan‐SE Version 2.0 (Lowe & Chan, 2016; Lowe & Eddy, 1997) with default parameters to identify tRNA genes, (b) the RNAmmer Version 1.2 (Lagesen et al., 2007) algorithm with the default parameters to identify rRNA genes (5.8S, 18S, and 28S), (c) nucleotide BLAST (Altschul et al., 1990) against miRBase release 22.1 (Kozomara & Griffiths‐Jones, 2014) with dust parameters and an e‐value of 0.00001. Filtering was based on identity and a query coverage of at least 90%.

### 2.10 Whole‐genome duplication analysis

Whole‐genome duplication events were predicted according to the distribution of synonymous substitutions per synonymous site (K) among paralogs. First, an all‐by‐all BlastP was run with the default parameters to scan all potential homologous gene pairs within any genome (*P. organensis*, *P. trichocarpa*, *S. purpurea*, *M. esculenta*, and *R. communis*; see Supplemental Table S1). The DupGen_finder pipeline (Qiao et al., 2019) was then run to identify WGD‐derived gene pairs on the basis of the MCScanX algorithm (Wang et al., 2012) bundled with the pipeline, and to calculate the K (Qiao et al., 2019) (see https://github.com/qiao‐xin/Scripts_for_GB).

To assess the genome similarities among related Malpighiales species we used the entirely sequenced and well‐annotated genomes of *P. trichocarpa, S. purpurea* (both Salicaceae), and *M. esculenta* and *R. communis* (both Euphorbiaceae) (Supplemental Table S1). MCScan was run with the default parameters according to the instructions [https://github.com/tanghaibao/jcvi/wiki/MCscan‐(Python‐version)]. Searching each genome for homologous groups of protein‐coding genes was based on the best five hits with the best nonself match in each target genome that met an e‐value threshold of 0.00001.

For constructing the matrix of average nucleotide identity (ANI) across coding regions, information was extracted from the BLAST output using Custom Perl scripts and the Malpighiales genomes available in Phytozome database Version 13 (Goodstein et al., 2012) (*P. trichocarpa*, *Populus deltoides*, *S. purpurea*, *M. esculenta*, *R. communis*, and *Linum usitatissimum* L.). *Arabidopsis thaliana* was used as the outgroup (Supplemental Table S1).

### 2.11 MADS‐box genes in *P. organensis*

By using the gene prediction pipeline combined with protein predictions output by Augustus Version 3.2.3 (Keller et al., 2011), we isolated the candidate *P. organensis* homologs in MADS‐box genes with the BlastP tool (Altschul et al., 1997) and *A. thaliana* sequences as baits. The MADS‐box sequences from *A. thaliana, P. trichocarpa*, and *Vitis vinifera* L. were obtained from the PlantTFDB database (Jin et al., 2017). Amino acid sequences were used for aligning the sequences on the basis of the L‐INS‐i algorithm in MAFFT Version 7 (Katoh & Standley, 2013). The alignments were recurrently inspected, then misaligned or nonaligned *P. organensis* sequences were examined further and either discarded or manually curated with Bioedit (Hall, 1999). The final alignment was fitted to the model with ModelFinder (Kalyaanamoorthy et al., 2017) implemented in IQ‐TREE Version 1.5.4 (Nguyen et al., 2015). The best fitting model, according to the Akaike criterion, was the Jones–Taylor–Thornton substitution model with empirical base frequencies and the discrete gamma model with four categories, which was used for phylogenetic reconstruction by maximum likelihood with IQ‐TREE Version 1.5.4. and 1,000 replicates in the bootstrap test.

### 2.12 Chloroplast and mitochondrial DNA assembly and annotation

GeSeq (Annotation of Organellar Genomes) (Tillich et al., 2017) with the default settings was used to predict *P. organensis* genes and identify sequence features, including chloroplast DNA protein‐coding gene sequences, rRNA, and tRNA, followed by manual correction for start and stop codons and intron positions in GenomeView software. All tRNA genes were further confirmed in tRNAscan‐SE (Lowe & Chan, 2016) and the ARAGORN online search server (Laslett & Canback, 2004). Pseudogenes were classified on the basis of the nucleotide losses or the presence of internal stop codons. Finally, the circular chloroplast genome map was constructed in OGDRAW (Greiner et al., 2019).

PacBio long reads were assembled in Canu Version 2.1.1 (Koren et al., 2017) with the default parameters. In total, 1,087,641 genomic reads (nuclear and organelle) were used in the assembly. Five mitochondrial contigs (built from 12,031 reads, i.e., 1.1% of the total genomic reads processed) were manually identified on the basis of the annotation and coverage similarity. Illumina short reads were used to polish the Canu contig mitochondrial sequences by read alignment and consensus extraction. Qiagen CLC Genome Workbench Version 8.5.1 was used to align the short reads against the first raw assembly. Cross_match (Machado et al., 2011) was used to close gaps in the scaffolds manually, generating two molecules, one circular (102,307 bp) and one linear (1,031,229 bp), with an average coverage of approximately 1200‐fold of the Illumina reads, 50‐fold of the Nanopore reads and 280‐fold of the PacBio reads.

For mitogenome annotation, the *P. edulis* genome (MT140634) (Yang & Wang, 2020) was used as the reference, following the protocol described for chloroplast DNA annotation. Gene sequences, intron–exon boundaries, and *trans*‐spliced events were manually curated by comparisons with annotated orthologs available in the plant mitochondrial genome database at NCBI (www.ncbi.nlm.nih.gov/genome/organelle). All other genetic elements were detected by manual BlastX and nucleotide BLAST searches against GeneBank, identifying the repeats in Blast2seq. Maps were constructed with the Circos Version 0.69‐9 visualization tool (Krzywinski et al., 2009) and manually adjusted with Inkscape (https://inkscape.org/).

## 3 RESULTS

### 3.1 The *P. organensis* karyotype

**FIGURE 2:**
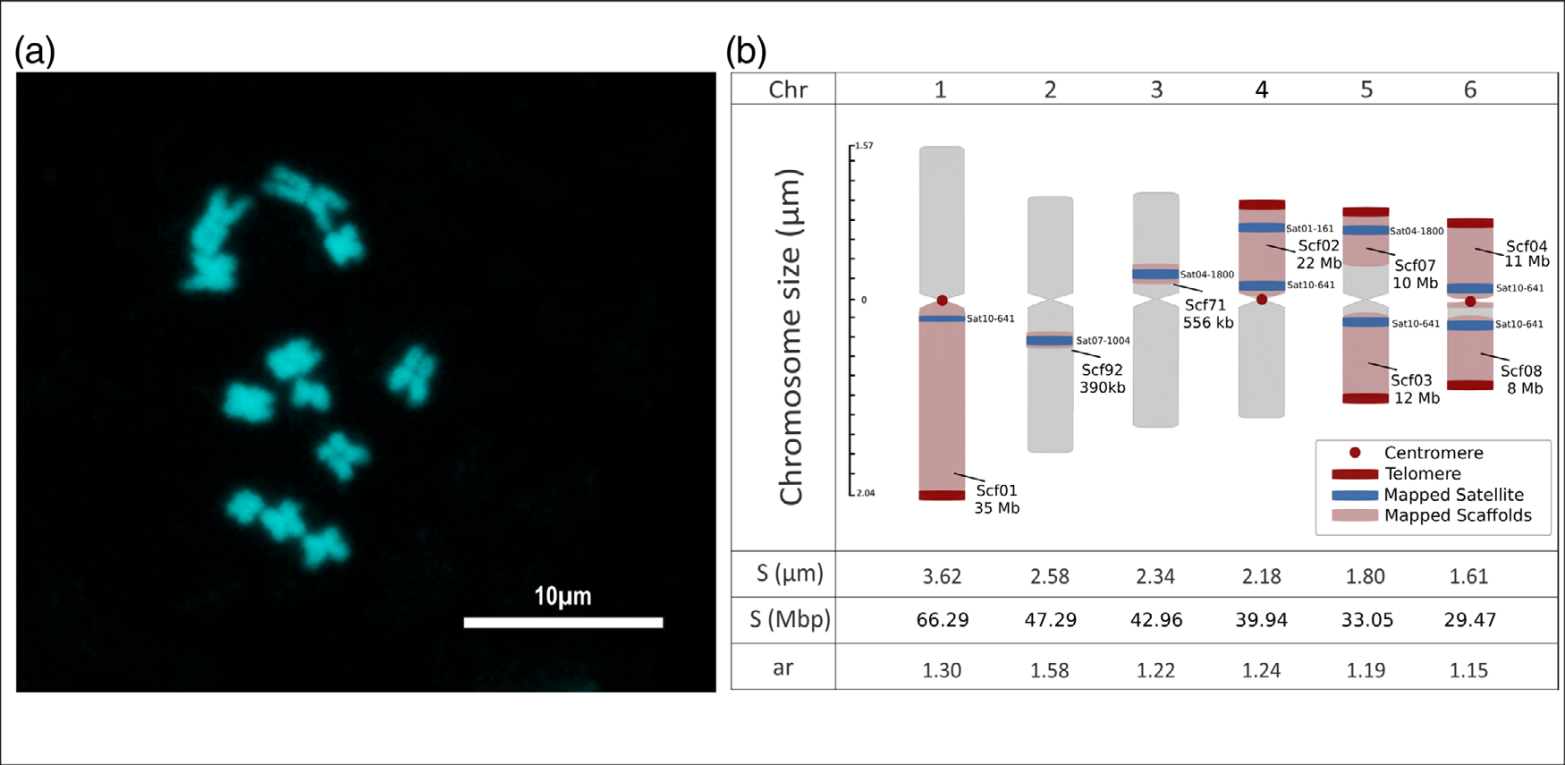
Partial integration of the genome sequences of *Passiflora organensis* into its chromosomes. (a) Mitotic metaphase showing *2n* = 12. (b) Ideogram showing the estimated size (S) of each chromosome, the arm ratios (ar), and the anchoring of the assembled pseudochromosomes and scaffolds (Sc) on each chromosome with the satDNAs indicated

Karyotype analysis of *P. organensis* revealed five pairs of metacentric chromosomes and one—the second largest—of submetacentric chromosomes (Figure 2a), ranging from approximately 3.62  to 1.61 μm in size (Figure 2b). We used satellite DNA sequences (Sader et al., 2021) as FISH probes for unambiguous identification of chromosome pairs and to facilitate contig and scaffold anchoring to chromosomes. As a result, satellite DNA PorSat07‐1004 was mapped in the long‐arm proximal region of chromosome 2, PorSat01‐161 in a large short‐arm heterochromatic block of chromosome 4, and PorSat04‐1800 was found in the short‐arm proximal region of chromosome 3 and within the short‐arm distal region of chromosome 5 (Figure 2b). Furthermore, when PorSat10‐641 was used as a probe, pericentromeric signals were observed in all chromosome pairs, but with lower abundance in chromosome 1.

### 3.2 Assembly and annotation of the *P. organensis* genome

We generated a total of 92 Gbp (PacBio and three Illumina sequencing libraries, Supplemental Table S2) to assemble a 259‐Mbp genome sequence in 482 contigs on 360 scaffolds with a guanine–cytosine content of 38.3% (Table 1, Supplemental Table S3). Genome sequence coverage was 160×. BioNano data were used to improve the assembly (Supplemental Table S4 and Supplemental Table S5). At least one end of six scaffolds (01, 02, 03, 04, 07, and 08) exhibited long telomeric repeats (AAACCCT)n (Figure 2b). By using dot‐plot approaches, we identified the centromeric and/or pericentromeric regions (which are difficult to tell apart) for two scaffolds (01 and 02), mapping the complete assembled sequences to the long and short arms of chromosomes 1 and 4, respectively. Three scaffolds (03, 06, and 10) had centromeric‐like repeats and another two (05 and the nonmapped scaffold 21) had interstitial telomeric repeats (Supplemental Figure S2, Supplemental Table S6).

**TABLE 1:** Statistics for the *Passiflora organensis* genome, including some comparisons with those of *P. edulis* (Xia et al., 2021)

Type	Value	*P. edulis* genome
Assembled genome size (bp)	259,301,974	1,327,182,440
Number of scaffolds	360	9
Longest scaffold	35,942,160 bp	204.53 Mb
Shortest scaffold	4,400 bp	112,417,078 bp
Number of scaffolds > Mb	38	9
Number of scaffolds >10 Mb	7	9
Scaffold N50 length^a^	8,258,683 bp	140.18 Mb
Scaffold L50 count^a^	9	5
Contig N50 length	2,458,705 bp	3.1 Mb
Guanine–cytosine content (%)	38.3	38.68
Number of protein‐coding genes	25,327	23,171
Average gene length	3,200 bp	–
Mean length of exons per gene	304 bp	–
Repeat content (%)	58.55	23.61

^a^ N50, the sequence length of the shortest contig at 50% of the total genome length; L50, the sequence length of the shortest scaffold at 50% of the total genome length.

The karyotype information, together with mapping of the satellite sequences by FISH and bioinformatic approaches, was used to integrate the scaffolds into *P. organensis* chromosomes. Eight of them have been mapped (01, 02, 03, 04, 07, 08, 71, and 92) (Figure 2; Supplemental Table S6), corresponding to 102.3 Mbp or ∼39% of the whole genome. K‐mer frequency analysis supported the *P. organensis* genome's diploid state, showing a heterozygosity rate of 81% (Supplemental Figure S3 and Supplemental Figure S4).

Note that we evaluated the possibility of using the available *P. edulis* genome sequence (Xia et al., 2021) to assist in assembling the *P. organensis* genome. However, because of structural chromosomal differences (number and size), it was not possible to anchor the *P. organensis* scaffolds onto fully assembled *P. edulis* chromosomes to reconstruct potential pseudomolecules.

Genome completeness assessment with Benchmarking Universal Single‐Copy Orthologs resulted in 1,587 (98.4%) of the expected 1,614 conserved plant orthologs (Supplemental Table S7). The LTR assembly index, a metric that evaluates genome continuity (Ou et al., 2018), was 12.19.

The combination of transcriptomic alignments and ab initio gene predictions allowed us to identify 25,327 genes (31% of the genome). These genes were identified on 233 scaffolds comprising 252 Mbp. The remaining 127 scaffolds 7 Mbp, with the sequence length of the shortest contig at 50% of the total genome length being 101 kbp) consisted of TE sequences. Approximately 21,000 genes were annotated for protein function, gene ontology assignment and InterPro domain. Annotation of noncoding and structural RNA genes revealed 492 tRNAs, at least 122 rRNAs (110 5.8S and six each of 18S and 28S rRNAs) and 83 miRNA genes.

We investigated the presence of genes potentially involved in the self‐incompatibility (SI) determining locus. *Brassica* sequences were used as a reference because of their well‐known system and their use in a previous investigation of *P. edulis* (Madureira et al., 2014). In total, 54 sequences were recovered from the *P. organensis* dataset, similar to SRK and SLG (Takayama & Isogai, 2005) (Supplemental Table S8). They included 14 SRKs (B_lectin‐SLG‐PAN_APPLE‐TM‐KD) and four SLGs (B_lectin‐SLG‐PAN_APPLE) with a typical domain architecture (Xing et al., 2013). All the others exhibited variant domain structures, which were assumed to correspond to precursors (degenerated products or functions still unknown) (Xing et al., 2013). Note the genome organization laid on Scaffold 6, which harbors a cluster of 10 genes (133,003 bp), including one SRK (scaffold6_gene573) and one SGL (scaffold6_gene572), as detected in *Brassica* (Takayama & Isogai, 2005). The role of these genes in the reproductive system of *P. organensis* requires further investigation.

### 3.3 Transposable element discovery

**FIGURE 3:**
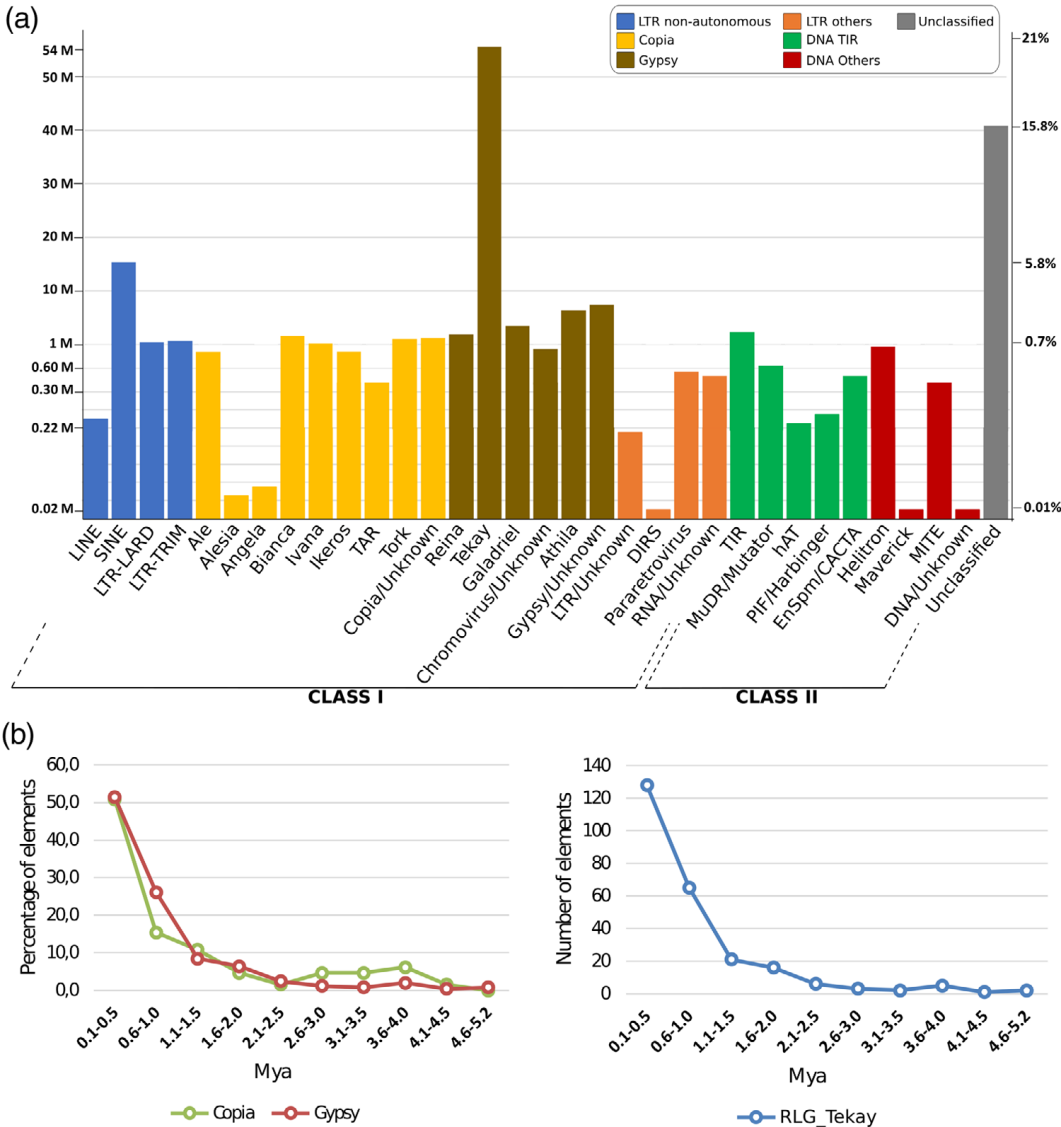
Transposable elements recovered in the *Passiflora organensis* genome. (a) The length (in Mbp) (on the left) and percentage occupied by each evolutionary lineage (on the right) were plotted on the *y*‐axis. (b) Estimated insertion times of the retrotransposon evolutionary lineages *Copia*, *Gypsy*, and *RLG_Tekay* in the *Passiflora organensis* genome

In total, 58.55% of the whole *P. organensis* genome consisted of repetitive elements (Figure 3a; Supplemental Table S9), most belonging to the LTR order (33.81%). Our analysis suggests a massive expansion of the LTR‐RT *Tekay* evolutionary lineage (*Gypsy* superfamily), representing up to 54 Mbp of the genome, accompanied by an expansion of long interspersed nuclear elements spanning ∼14 Mb. A small fraction (2.79%) of the *P. organensis* genome harbored DNA transposons, and unclassified TEs accounted for up to 15.82%.

Insertion time analysis of the *Copia* and *Gypsy* superfamilies indicated that nearly 50% of the TEs had been inserted into the *P. organensis* genome within the past 0.5 million years, whereas others had appeared between 0.6 and 5.2 mya. Moreover, the insertion time of the *Tekay* lineage, the most predominant of all the TEs, suggests the recent activity of these elements, since the majority appeared from 0.1 to 0.5 mya (Figure 3b).

Some copies of nonautonomous TEs were also identified, including large retrotransposon derivatives and terminal repeat retrotransposons in miniature from the LTR order, and miniature inverted repeat transposable elements derived from DNA transposons. Some are known to lack domains and also to interact with host genes, incorporating fragments or even entire sequences. In fact, nonautonomous *P. organensis* TEs harboring gene sequences were found. Interestingly, all DNA transposons in the Helitron order appeared to incorporate gene fragments, the majority in domains related to resistance genes (Supplemental Table S10).

### 3.4 Whole‐genome duplication analysis

**FIGURE 4:**
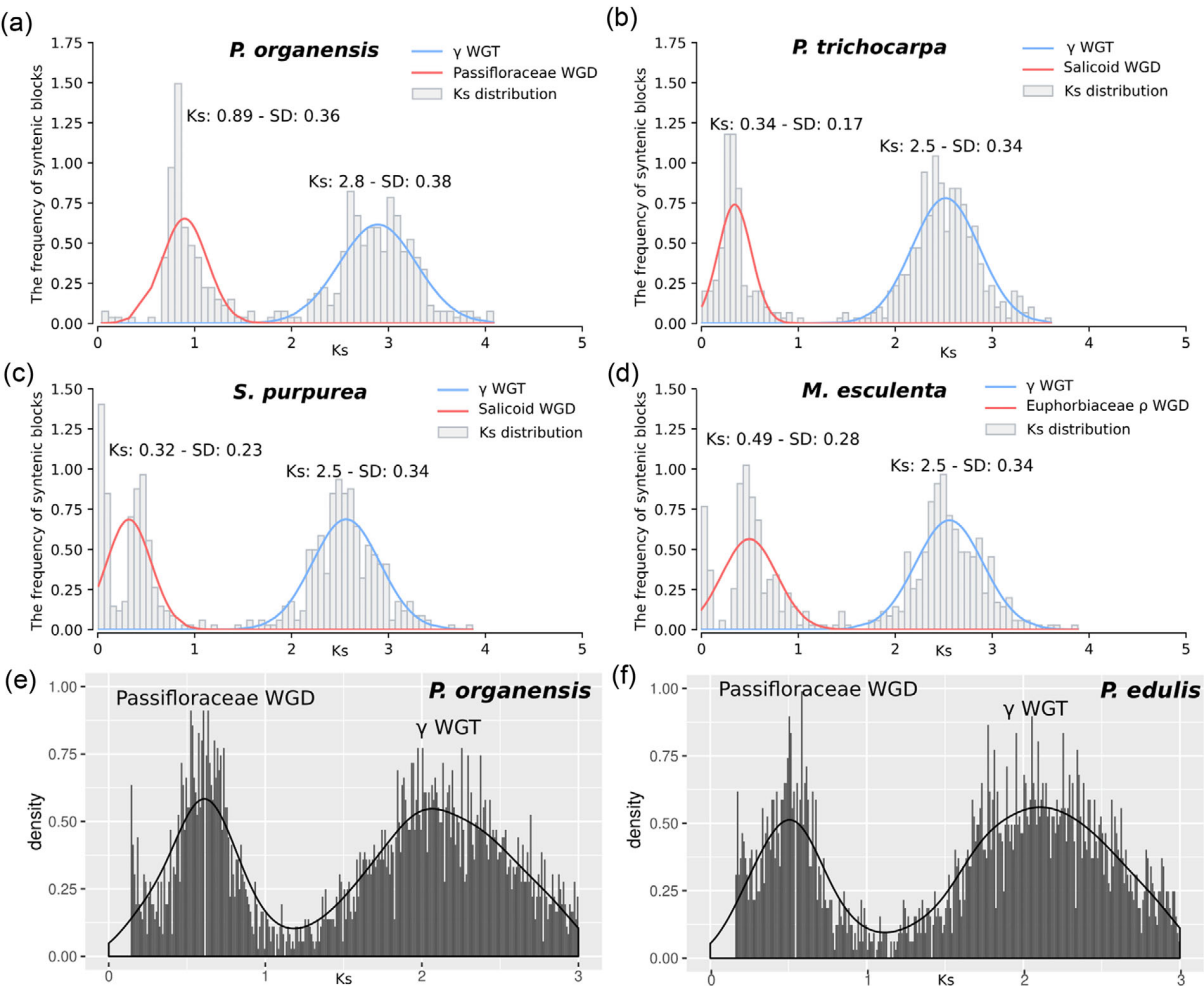
Whole‐genome duplication (WGD) analysis. The distribution of synonymous nucleotide substitution rates (K) defining the duplication of large chromosomal regions in the genomic data of (a) *Passiflora organensis*, (b) *Populus trichocarpa*, (c) *Salix purpurea*, and (d) *Manihot esculenta*. Distribution of K rates based on transcriptomic data from (e) *P. organensis* and (f) *Passiflora edulis*. All species share the ancient genome triplication event [γ whole‐genome triplication (WGT)] associated with the early diversification of the core eudicots

The distribution of pairwise synonymous substitution rates across the corresponding duplicated genes (paralogs) in conserved genomic blocks was studied in *P. organensis* and the Malpighiales *P. trichocarpa*, *S. purpurea* (both Salicaceae), and *M. esculenta* (Euphorbiaceae). These are related species with entirely sequenced and well‐annotated genomes (Supplemental Table S1). Synonymous nucleotide substitution rate‐based analysis (Cai et al., 2019) identified WGDs in all species (Figure 4a–d). The Ks distribution were also examined in the transcriptomic data from *P. organensis* (subgenus *Decaloba*) and a combination of three plants of the sour passionfruit (*P. edulis*, subgenus *Passiflora*) (Cai et al., 2019). These species share the ancient genome triplication event (γ whole‐genome triplication), exhibiting a second peak that corresponds to a second shared WGD event (Figure 4e,f) beginning in the Eocene, according to Cai et al. (2019).

**FIGURE 5:**
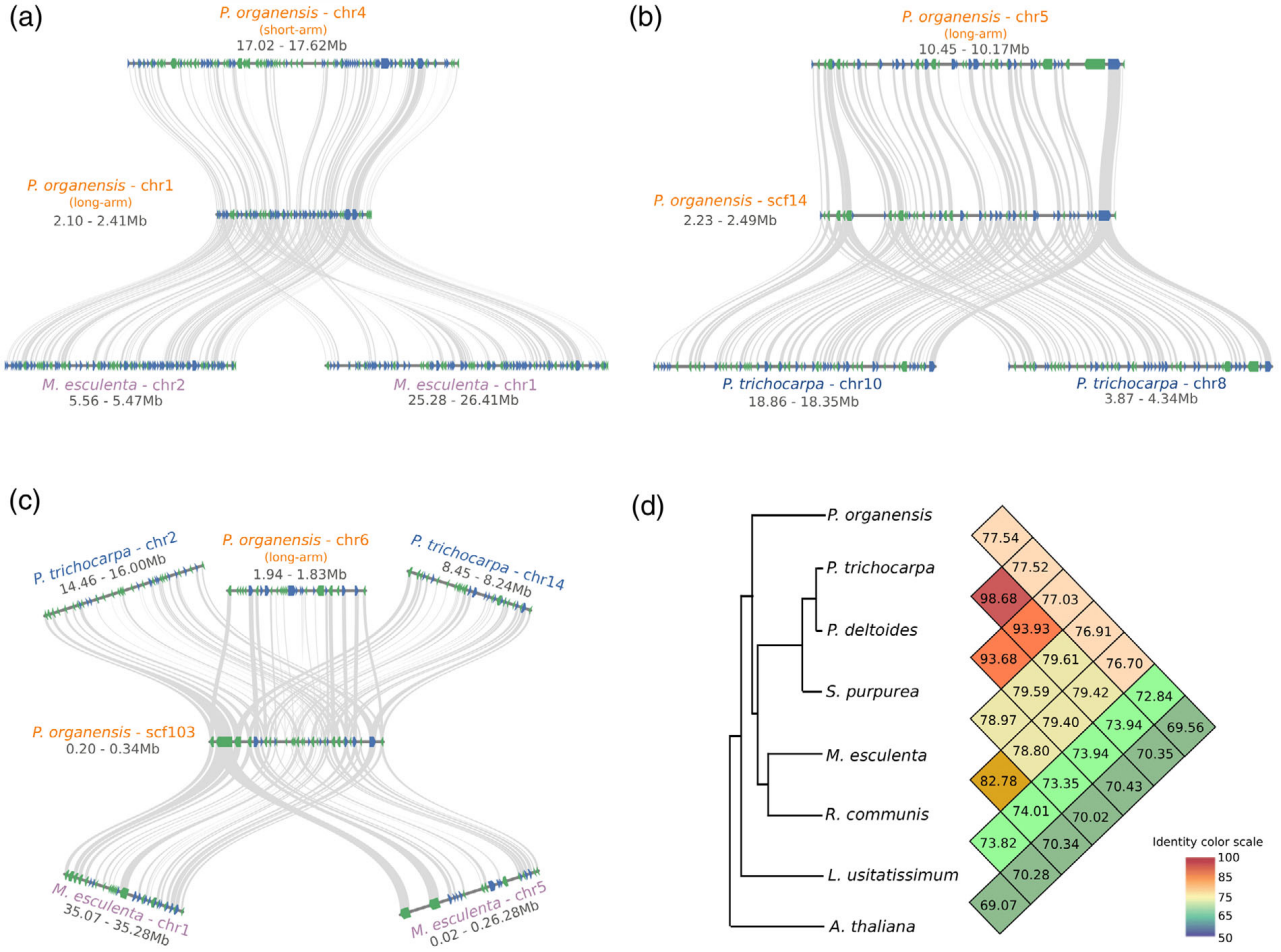
Microsynteny and comparative analysis of *Passiflora organensis* and other Malpighiales, showing different patterns of gene retention and loss in different segments of the same genome, and across the genome segments of each species. (a, b) Exclusive and shared microsyntenic segments in the genomes of *P. organensis* and *Manihot esculenta*, and in *P. organensis* and *Populus trichocarpa*: (a) mycrosinteny between *P. organensis* and *M. esculenta*; (b) mycrosinteny between *P. organensis* and *P. trichocarpa*. (c) Shared microsyntenic segments in the genomes of *P. organensis, M. esculenta*, and *P. trichocarpa*. (d) Average nucleotide identity (ANI) and neighbor‐joining tree based on the coding regions of all sequenced Malpighiales genomes available in public databases; *Arabidopsis thaliana* was the outgroup

As expected, the compared Malpighiales species exhibited extensive blocks of microsynteny, showing distinct gene retention patterns, fractionation, and alternative deletions of duplicated genes. Some of these rearrangements are shown in Figure 5a–c. In general, the Malpighiales analyzed were collinear in regard to gene orders, supporting the idea of a common ancestor and distinct levels of genome‐wide reorganization in each lineage after Malpighiales diversification.

Moreover, ANI between coding regions revealed higher levels of coding region ANI (> 93%) in different Salicaceae species (e.g., *P. trichocarpa*, *P. deltoides*, and *S. purpurea*; Figure 5d). In contrast, related Euphorbiaceae, such as *M. esculenta* and *R. communis*, exhibited lower coding region ANI (∼82%). Unsurprisingly, the Salicaceae, Euphorbiaceae, and Passifloreacea species were generally far more similar to each other in terms of coding regions ANI than individual species compared with *L. usitatissimum* (Linaceae), corroborating their phylogenetic distance.

### 3.5 Orthogroups and GO enrichment analysis

**FIGURE 6:**
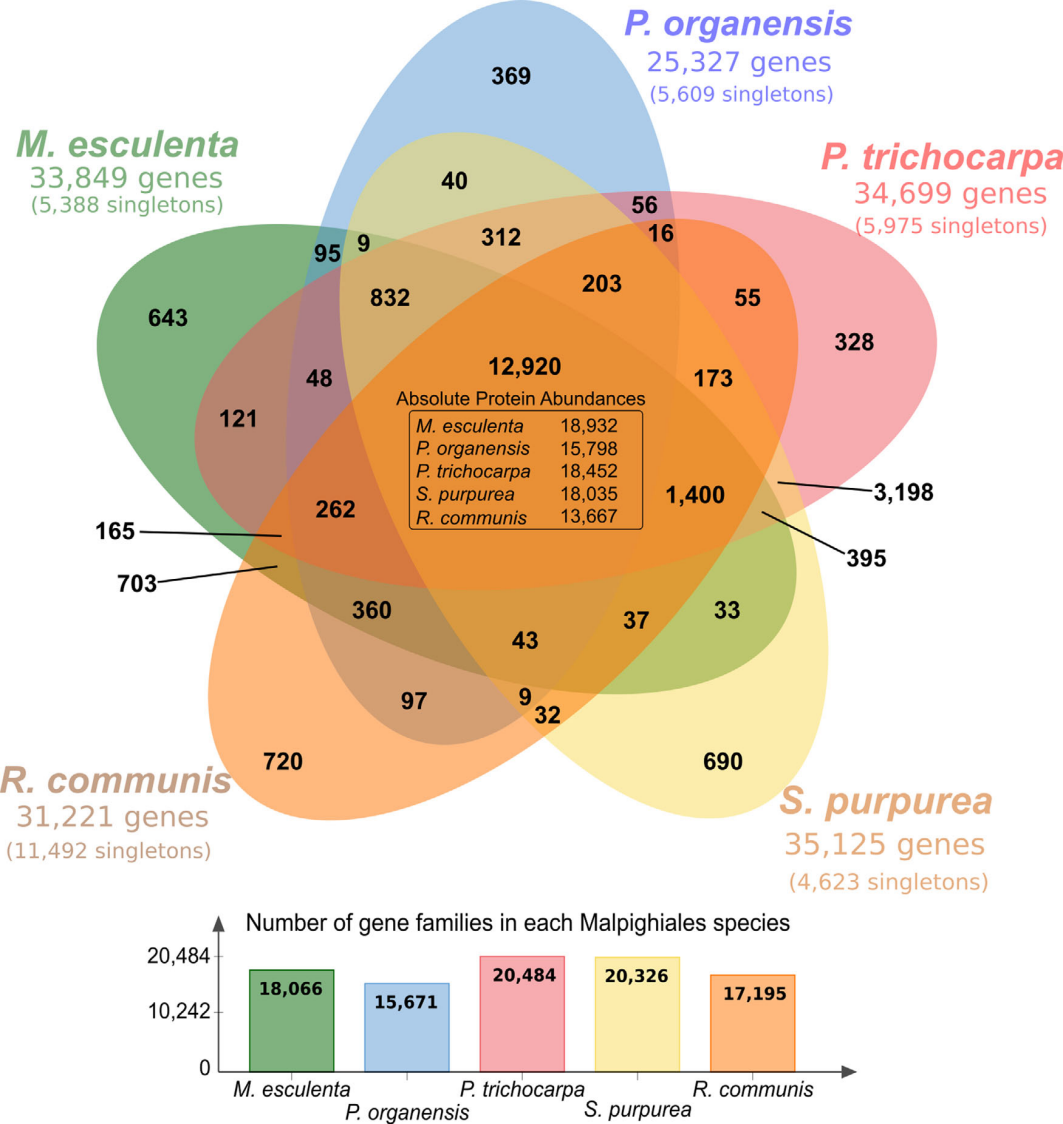
Venn diagram showing the distribution of orthologous gene families among the Malpighiales *Passiflora organensis* (Passifloraceae), *Populus trichocarpa*, *Salix purpurea* (both Salicaceae), *Ricinus communis*, and *Manihot esculenta* (both Euphorbiaceae)

We used Ortho‐MCL to assess orthologous families in each genome and GO enrichment patterns in the complete sequenced genomes of *P. organensis*, *P. trichocarpa*, *S. purpurea*, *M. esculenta*, and *R. communis*. In total, 25,327 coding genes were predicted. *Passiflora organensis* retains 5,609 singletons and 15,671 gene families. Of this total, *P. organensis* shared 12,920 gene families with all the genomes compared (Figure 6), and 981 genes were unique and grouped in 369 families (Supplemental Table S11). The number of members in each family ranged from 2 to 15 genes. The largest family belonged to a hypothetical protein (Cluster 316), followed by a family of proteins (Cluster 415) with DNA‐binding domains (GO 0003677).

The genomes analyzed shared enriched GO terms mostly related to development, defense, and molecular events such as transcription, translation, and signal transduction (Figure 6). Each species had a particular set of terms assigned for protein‐encoding genes in a given functional category. For *P. organensis*, the enriched terms were the biological process medium‐chain fatty acid metabolic process (GO:0051791) and the molecular function glutathione transferase activity (GO:0004364), encompassing six proteins. The former may be relevant for seed oil composition studies in *Passiflora* (see Krist, 2020). Glutathione transferase activity has some relevance to various biological processes, from plant development to defense against biotic and abiotic stresses (Gallé et al., 2019). For the complete list of GO annotations, see Supplemental Table S12.

We also investigated whether selection pressure exerted bias on the distribution of genes according to functional categories, taking account of WGD and other duplication events (dispersed, proximal, and tandem). Most of the genes in each of the function categories to which the GO terms had been assigned were duplicated in large‐scale events (WGD). Only functions related to circadian processes (rhythmic process, GO:0048511; circadian rhythm, GO:0007623) were biased towards local tandem duplication events (Supplemental Table S12).

### 3.6 The *P. organensis* MADS‐box gene family

**FIGURE 7:**
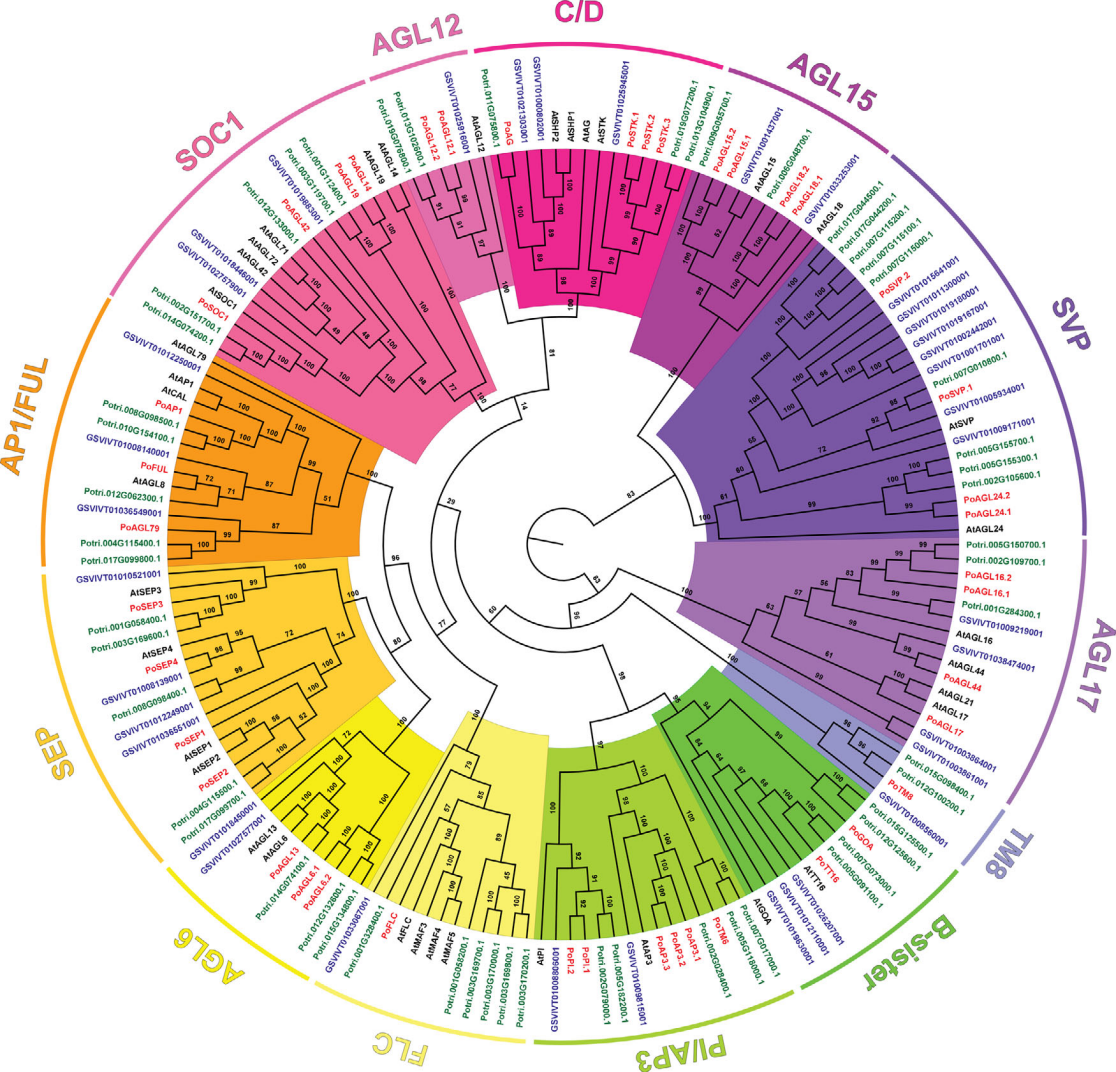
Phylogenetic analysis of MADS intervening keratin‐like and *C*‐terminal (MIKC)‐type MADS‐box genes in *Passiflora organensis*, *Arabidopsis thaliana*, *Populus trichocarpa*, and *Vitis vinifera*. The sequences were separated into groups: C and D gene classes (C/D), agamous‐like genes (AGL), Flowering Locus c (FLC), sepallata (SEP), apetala 1/fruitfull (AP1/FUL), suppressor of overexpression of CO 1 (SOC1), tomato mads‐8 (TM8), short vegetative phase (SVP), B‐sister (the sister group of the B genes), and pistillata/apetala 3 (PI/AP3). Sequences prefixed Po relate to *P. organensis*, At to *A. thaliana*, Potri to *P. trichocarpa*, and GSVIV to *V. vinifera*. The numbers at the nodes are the bootstrap test results

As an example of how expansions and contractions in gene families have affected the structure of the *P. organensis* genome, we compared it with the MADS‐box family members in *A. thaliana*, *P. trichocarpa*, and *V. vinifera* (Figure 7). The MADS‐box family of transcription factors is involved in many important developmental processes, and they are well‐known for their role in phase transition and establishing the identity of floral organs [reviewed by Kramer (2019)]. We chose this gene family because some of its members have already been studied in detail by our group and might have important biological and evolutionary roles in *Passiflora*, especially with regard to reproductive development (Cutri & Dornelas, 2012; Scorza et al., 2017; Silveira et al., 2016). We initially found 104 putative MADS‐box family members in *P. organensis*. After manual sequence curation, we ended up with 75 complete sequences that most probably represent the total set of family members in *P. organensis*. Among these sequences, 33 belong to the M‐type (Type I) and 42 to the MADS intervening keratin‐like and *C*‐terminal (MIKC)‐type (Type II). By comparing these sequences with those found in other plant genomes, we were able to infer expansions and contractions within specific subfamilies (see Supplemental Figure S5 and Supplemental Figure S6; Supplemental Table S13 and Supplemental Table S14).

The accuracy of identification of MADS‐box family members is shown in Figure 7. The bootstrap values of branches containing putative *Passiflora* MADS‐box family members and recognized MADS‐box members from other plant species (including the model species *A. thaliana*) ranged from 75 to 100%, confirming the accuracy of paralog and ortholog identification.

We observed a drastic reduction in the number of members belonging to the M‐type subfamily compared with *A. thaliana* and *P. trichocarpa* (Supplemental Figure S6, Supplemental Table S13, Supplemental Table S14). Because of their acknowledged importance in modulating plant development, we focused on the MIKC‐type members. The presence of a sequence belonging to TM8 is also noteworthy; this sequence is missing from the *A. thaliana* genome, but present in many other species, including *P. trichocarpa* and *V. vinifera* (Supplemental Figure S6, Supplemental Table S13, Supplemental Table S14). Nonetheless, the group that exhibited the largest expansion within the MIKC‐type MADS‐box genes was PI/AP3, with six members in *P. organensis* but only two in *A. thaliana* and *V. vinifera* (Supplemental Table S13).

### 3.7 Chloroplast and mitochondrial genomes

A typical chloroplast DNA circular molecule was recovered with a quadripartite structure spanning 144.959 Mbp. It has 37% guanine–cytosine content and encodes a total of 104 unique genes (Supplemental Table S15, Supplemental Figure S7), 70 of which are protein coding genes, four of which are rRNA genes (4,5S, 5S, 16S, and 23S) and 30 of which are tRNA genes. The *P. organensis* chloroplast genome contains rearrangements that occurred throughout the *Decaloba* subgenus's evolution, such as the contraction of inverted repeat regions, and gene and intron losses (Supplemental Figure S8).

**FIGURE 8:**
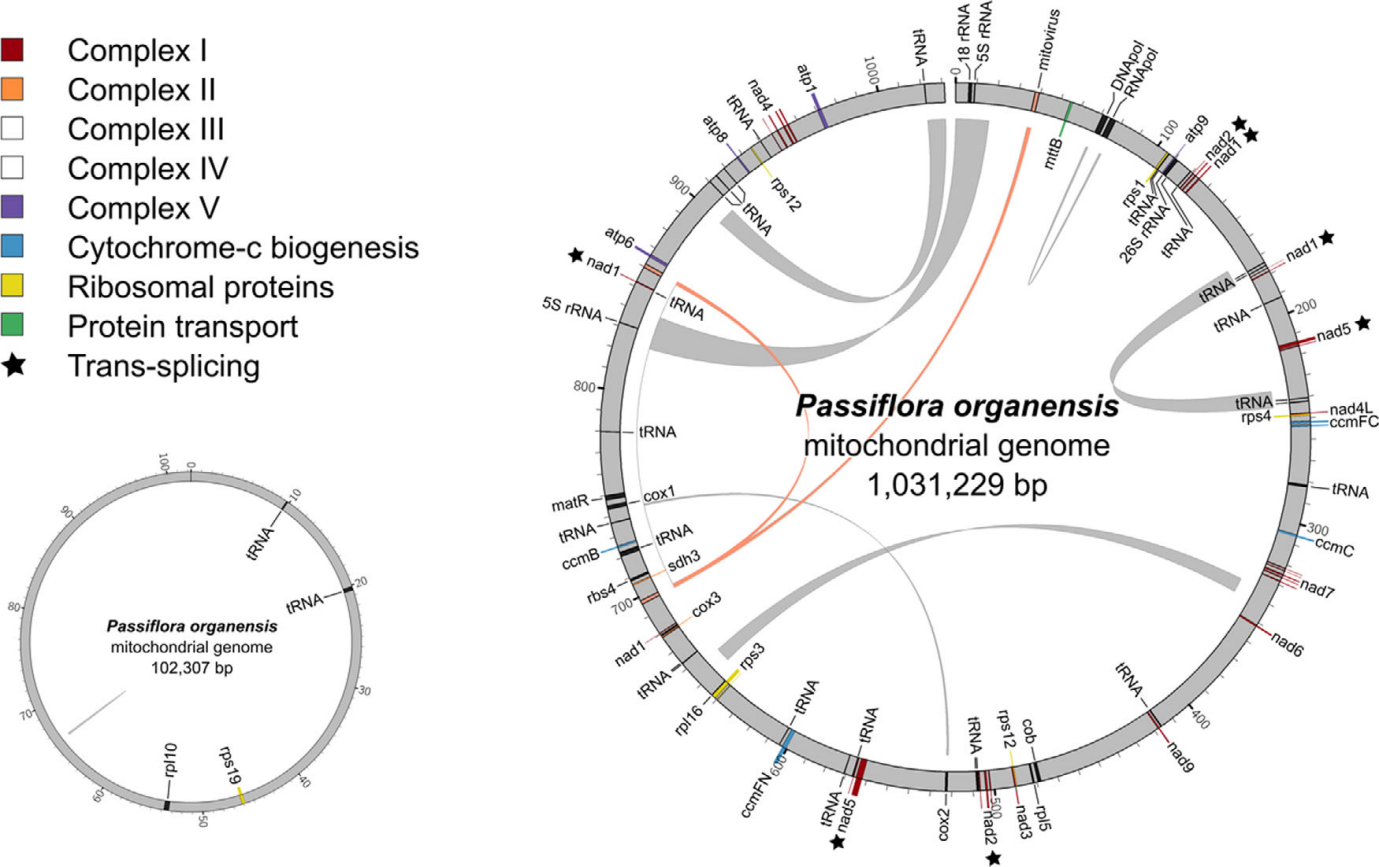
Schematic representation of the mitochondrial circular (left) and linear (right) molecules of *Passiflora organensis*. Their main features and gene organization are indicated by bars with gene names, tRNA, and rRNA. Repeats are represented as ribbons inside the circles. For the linear molecule, the ribbons link sequences longer than 800 bp sharing >75% identity. For the circular molecule, only one ribbon links a repeat of 200 bp sharing 89% identity. Stars after the gene name denote genes whose transcripts require *trans*‐splicing

We assembled two mitochondrial DNA molecules confirmed throughout with long reads (PacBio and Nanopore) and pairs of short Illumina reads. The linear sequence represents the master mitochondrial DNA molecule comprising 1,031,229 bp (45% guanine–cytosine content), harboring at least one full copy of most canonical mitochondrial protein‐encoding genes (32), rRNA genes (two full copies of 5S, and one each of 18S and 26S), and 30 tRNA genes. These genes were interspersed with three mitoviruses comprising a repetitive region of ∼1,800 bp, besides relics of other mobile and repetitive elements (Figure 8, Supplemental Table S16, Supplemental Table S17). The mitogenome also exhibited interspersed sequences of chloroplast DNA. The various repetitive sequences were interspersed within intergenic regions, providing potential sites for rearrangement. In Figure 8, we have highlighted regions of this kind with more than 800 bp and 75% identity.

The genes encoding subunits of the mitochondrial membrane respiratory chain nicotinamide adenine dinucleotide hydrogen (NADH) dehydrogenase Complex I, Nad1 (NADH–ubiquinone oxidoreductase chain 1), Nad2 (NADH‐ubiquinone oxidoreductase chain 2), and Nad5 (NADH‐ubiquinone oxidoreductase chain 5), exhibited a combination of introns, some of which required *trans*‐splicing reactions (Figure 8, Supplemental Table S16, Supplemental Table S17).

The second molecule was circular, comprising 102,307 bp and harboring full versions of two canonical genes missing on the linear molecule (*rsp19*, *rpl10*), besides extra lysine and methionine tRNA. The repeats in the circular version were smaller than 200 bp. In Figure 8, we have highlighted a unique region of 200 bp with an identity higher than 75%.

## 4 DISCUSSION

There are many studies on the attributes of *Passiflora* species (Ingale & Hivrale, 2010; de Castro et al., 2018; Cauz‐Santos et al., 2020; Krist, 2020) but they have been severely limited by the lack of available sequence data. Even an initial draft genome assembly opens up a wide range of possibilities that would simply not be feasible without a reference (Worley et al., 2017). The availability of a draft genome for the wild *P. organensis* (subgenus *Decaloba*) will provide a valuable resource for genetic, genomic, functional, and evolutionary studies of *Passiflora*.

By using a hybrid approach combining Illumina and Pacbio sequencing technologies and Bionano optical maps, we were able to generate a 259‐Mbp genome, consistent with the estimate obtained by flow cytometry (Yotoko et al., 2011) and approximately 5.4× smaller than the genome of the purple form of *P. edulis* (Xia et al., 2021). As expected, we identified some conflicts when attempting to correctly map the scaffolds onto chromosome arms, and cytogenetic markers were fundamental for resolving some of them. Eight scaffolds were integrated, accounting for ∼102 Mbp (Figure 2, Supplemental Table S6). Centromeres were also located in chromosomes 1, 4, and 6; telomeres in chromosomes 1 and 4 (one end), and 5 and 6 (both ends). Combining these approaches significantly improved the final assembly.

A good‐quality assembly was obtained and confirmed by parameters, such as high consensus quality (99.98%), a LTR assembly index of 12.19, and complete conserved plant orthologs reaching 98.4%, a value also obtained for other assemblies (Wei et al., 2020; Zhang et al., 2020).

Herein we present the first complete set of genes from a wild *Passiflora* species, including the MADS‐box family of transcription factors, putative genes related to self‐incompatibility, and so on. This set of genes could significantly assist teams working on *Passiflora*, facilitating searches for specific genes and different kinds of analysis, such as the genetic changes associated with domestication and comparative studies on other species, including the purple form of *P. edulis* recently made available (Xia et al., 2021). Some of the most abundant gene families in this domesticated species are implicated in the defense response and terpene synthase activity (see Supplemental Figure S9), and many terpenoids exhibit potent toxicity and serve as core components of chemical defenses against herbivores, insect pests, and microbial pathogens.

Consistent with the reproductive mechanisms of several *Passiflora* that behave as outcrossing species, a high level of heterozygosity (81%) was found. Some attempts to self‐cross *P. organensis* were unproductive (personal communication, M.C. Dornelas, 2020), suggesting that it is self‐incompatible. Self‐incompatibility is one of the most important genetic mechanisms for impeding self‐fertilization but is poorly understood in *Passiflora*. It has been reported that *Passiflora* has a homomorphic SI system controlled by sporophytic and gametophytic mechanisms (Madureira et al., 2014). We investigated two of the SI reaction determinants (female) in the SI locus described for Brassicaceae [reviewed in Takayama & Isogai (2005)]. We searched for the SRK gene, which regulates the SI response in the stigma, and SLG, which boosts SI response efficiency (Takasaki et al., 2000) via a modified approach first proposed for *P. edulis* (Madureira et al., 2014). Fifty‐four proteins in *P. organensis* have domains commonly detected in the proteins encoded by the S‐locus (Xing et al., 2013). We detected a cluster of genes in *P. organensis* with the structural characteristics of the S‐locus, on the basis of the proximity of the SRK and SLG candidate genes. Identification of the S‐locus would be the first step towards elucidating SI in *Passiflora*. On the basis of the sequences identified herein, it would be possible to detect SI genes in commercialized species e.g., *P. edulis* and *Passiflora alata* Curtis, which could help in breeding improved cultivars.

Transposable elements are important drivers of plant genomes’ structure and evolution. For the first time, all TE copies in a *Passiflora* species were identified. Previous studies have identified a collection of complete TEs in *P. edulis*, but only for a gene‐rich fraction of the genome (∼1%) (Costa et al., 2019). In line with the majority of plant genomes (Orozco‐Arias et al., 2019), the most frequent elements in *P. edulis* and *P. organensis* were from the LTR order, particularly the *Tekay* lineage, a sign of possible activity. Intriguingly, long interspersed nuclear elements have significantly proliferated in *P. organensis*, although they are less probable in plants (Wicker et al., 2007). In addition, insertion time analysis suggested that LTR‐RTs were recently inserted into the *P. organensis* genome, a pattern similar to that found in *P. edulis* (Costa et al., 2019), possibly indicating that full‐length LTR‐RT elements have recently become active. Note that repetitive sequences are very difficult to assemble and, consequently, can result in highly fragmented assemblies. This made the challenge we faced even greater (Michael & VanBuren, 2015).

In a comparative analysis of the MADS‐box family, we identified 75 complete sequences, 33 of the M‐type (Type I) and 42 of the MIKC‐type (Type II). A drastic reduction in the number of members belonging to the M‐type subfamily was observed. This contraction was previously reported to occur in *Vitis* (Grimplet et al., 2016), but its biological implications remain unexplained. A sequence belonging to the TM8 group was also identified. This sequence is missing from the *A. thaliana* genome but is present in many other species, including *P. trichocarpa* and *V. vinifera* (Supplemental Figure S6, Supplemental Table S13). The biological role of TM8 has been investigated in tomato (*Solanum lycopersicum* L.), where it functions in fruit development (Daminato et al., 2014).

Finally, the PI/AP3 group showed the largest expansion within the MIKC‐type MADS‐box family (Supplemental Table S13). Expansion of the PI/AP3 group has been reported in Orchidaceae and Zingiberaceae (Bartlett & Specht, 2010; Mondragón‐Palomino & Theißen, 2008). Both plant groups exhibit floral structures that are sui generis (such as the labellum in Orchidaceae and the petaloid stamens in Zingiberaceae). These floral structures are considered evolutionary novelties and their appearance has been correlated with duplications followed by neofunctionalization of genes in the PI/AP3 group (Gioppato & Dornelas, 2019; Hernández‐Hernández et al., 2007; Kim et al., 2004; Rijpkema et al., 2010). Accordingly, the corona filaments in the flowers of *Passiflora* species are also considered sui generis floral organs or petaloid stamens (Bernhard, 1999; Hemingway et al., 2011), and it is tempting to speculate that the origin of the *Passiflora* corona might be related to the expansion of the PI/AP3 group of MADS‐box genes.

*Passiflora organensis* has 2*n* = 12, as do most species in the *Decaloba* subgenus (De Melo & Guerra, 2003; Hansen et al., 2006; Sader, Amorim, et al., 2019). Nevertheless, apart from the shared γ whole‐genome triplication in eudicots, *P. organensis* shares a WGD event with *P. edulis* dated to the Eocene (Cai et al., 2019), which coincides with the origin of the *Passiflora* genus around 42.9 mya (Sader, Amorim, et al., 2019). However, a recently published Ks analysis showed that two WGD events occurred 65 and 12 mya, which may have contributed to the large genome size of *P. edulis* (∼1341.7 Mb) (Xia et al., 2021). Further research is required to resolve these discrepancies.

Thus, in contrast to what we proposed earlier (Sader, Amorim, et al., 2019), the genomic data support the idea of *x* = 12 for *Passiflora* (Hansen et al., 2006), dropping to *n *= 6 by descending dysploidy in *P. organensis*. The drop in chromosome number back to *n* = 6 after the ancestral *Passiflora* WGD was accompanied by a reduction in genome size, resembles the genome repatterning observed in *A. thaliana* (Lysak et al., 2006). These rearrangements are supported by the discovery of interstitial telomeric sequences in the two scaffolds. On the basis of a probabilistic model [see Mayrose et al. (2010) for details] that assumes chromosome gains and losses, the predicted base chromosome number was *x* = 12, with a probability of 0.98 (Mayrose et al., 2010). The only possible type of event that would explain the chromosome numbers in the subgenera *Decaloba* is descending dysploidy, as specified herein. Differences in chromosome number and genome size between *P. edulis* and *P. organensis* are associated with important genomic rearrangements, shown here by a comparative analysis that indicated wide macroscale discrepancies and conserved microscale regions (Supplemental Figure S9A,B).

In land plants, the chloroplast DNA is a small, circular molecule but is nevertheless a vital one because it codes for some 100 proteins that, in combination with nuclear‐encoded proteins, play a part in important metabolic processes such as photosynthesis (Kleffmann et al., 2004; Sugiura, 1992). Despite the highly conservative nature of chloroplast DNA in angiosperms, the chloroplast genome of *P. organensis* exhibits rearrangements following the highly dynamic evolution undergone by the chloroplast DNA of *Passiflora* species (Cauz‐Santos et al., 2017; 2020; Shrestha et al., 2019). *Passiflora organensis* is placed in the *Decaloba* subgenus, which encompasses a high number of rearrangements already reported for Passifloraceae, with a combination of structural inversions, gene/intron losses, and variations in inverted repeat length caused by inverted repeat expansions and contractions, including the loss of an entire inverted repeat region in some species (Cauz‐Santos et al., 2020; Shrestha et al., 2019).

Finally, we explored multiple assemblies of the mitochondrial genome with a combination of long and short reads to capture a sequence that would best represent the mitogenome of *P. organensis*. As reported in a study on lettuce (*Latuca* spp.) species (Kozik et al., 2019), variants of mitogenome sequences can be recovered in a whole genome sequencing project with long reads. We presented the two molecules assembled in this study, conclusively confirmed by short pair‐end Illumina reads and long Pacbio and Nanopore reads. The *P. organensis* mitogenome exhibits the canonical genes essential for respiratory chain functions and protein synthesis, including rRNA and tRNA. However, the complete set must include the two molecules assembled, one larger linear sequence of 1,031,229 bp and one small circular molecule of 102,307 bp. Despite numerous attempts, we did not find any reads combining the two molecules. The fact that there is more than one molecule in plant mitochondria comes as no surprise, since some authors have described one or more molecules, both circular and linear (Kozik et al., 2019). The *P. organensis* mitogenome is among the largest genomes assembled to date compared with other species (Wynn & Christensen, 2019). Only ∼5% of the sequence coded for proteins, rRNA, and tRNA. The remaining sequences were immigrants from the chloroplast and other genetic elements, most of which were not found in the *P. edulis* mitogenome (Yang & Wang, 2020).

One of these elements found in the *P. organensis* mitogenome consisted of three copies of an RNA‐dependent RNA polymerase of mitoviruses detected in many plant mitochondrial DNAs (Bruenn et al., 2015; Nibert et al., 2018). They are called nonretroviral endogenized RNA virus elements. These are simple viruses carrying only one RNA‐dependent RNA polymerase; they are mostly of fungal origin and were potentially acquired by mitogenomic horizontal transfer. Mitogenomes in plant pathogenic fungi are associated with a reduction in virulence towards their hosts. The role of such molecules in plant mitogenomes is still elusive (Nibert et al., 2018). Sequences related to mitoviruses were missing from the published version of the *P. edulis* mitogenome. Note that, in addition to variations in mitogenome intergenic regions, gene organization and the presence of introns involving both *cis*‐ and *trans*‐splicing were remarkably conserved. The number of large repeats merits further investigation since they may explain recombination events, the size of plant mitochondrial genomes, the recruitment of extra elements, and their contribution to the plant's evolution (Kozik et al., 2019; Wynn & Christensen, 2019).

## CONFLICT OF INTEREST

The authors declare that they have no competing interests. Mendelics Análise Genômica provided support only in the form of a salary for J.P.K., but had no role in study design, data collection and analysis, the decision to publish, or preparation of the manuscript.

## AUTHOR CONTRIBUTIONS

Zirlane Portugal Costa: formal analysis, investigation, methodology, writing—original draft. Alessandro Mello Varani: data curation, formal analysis, methodology, writing—original draft. Luiz Augusto Cauz‐Santos: data curation, formal analysis, investigation, methodology, resources, writing—original draft. Mariela Analía Sader: investigation, methodology, writing—original draft. Helena Augusto Giopatto: formal analysis, investigation. Bruna Zirpoli: investigation. Caroline Callot: investigation. Stephane Cauet: investigation. Willian Marande: data curation, methodology. Jessica Luana Souza Cardoso: formal analysis. Daniel Guariz Pinheiro: formal analysis, methodology. João Paulo Kitajima: data curation, formal analysis, methodology. Marcelo Carnier Dornelas: conceptualization, formal analysis, funding acquisition, supervision, writing—original draft. Andrea Pedrosa Harand: formal analysis, investigation, resources, supervision, writing—original draft. Helene Berges: conceptualization, supervision. Claudia Barros Monteiro‐Vitorello: formal analysis, investigation, methodology, writing—review and editing. Maria Lucia Carneiro Carneiro Vieira: conceptualization, funding acquisition, supervision, writing—review and editing

## Supporting information

Supplemental Figure S1. Steps in the assembly of the genome
Supplemental Figure S2. Dot‐plot alignment of the scaffolds of chromosome arms
Supplemental Figure S3. K‐mer profile plot of the genome
Supplemental Figure S4. Smudgeplot profile for the genome
Supplemental Figure S5. Phylogenetic analysis of M‐type MADS‐box genes
Supplemental Figure S6. Comparative representation of the MADS‐box gene family
Supplemental Figure S7. *Passiflora organensis* chloroplast DNA
Supplemental Figure S8. *Passiflora* chloroplast genome rearrangements
Supplemental Figure S9. Comparative analysis of *P. organensis* and Malpighiales species
Supplemental Table S1. Genome data from Malpighiales species
Supplemental Table S2. Statistics for the *P. organensis* genome
Supplemental Table S3. Statistics for *P. organensis* assembly and annotation
Supplemental Table S4. *Passiflora organensis* bionano data
Supplemental Table S5. *Passiflora organensis* bionano assembly
Supplemental Table S6. Number and length of assembled scaffolds
Supplemental Table S7. Evaluation of *P. organensis* assembly
Supplemental Table S8. *Passiflora organensis* SI locus
Supplemental Table S9. Transposable elements in *P. organensis*
Supplemental Table S10. Fragments of genes identified in transposons
Supplemental Table S11. Proteins unique to *P. organensis*
Supplemental Table S12. Gene Ontology enrichment data
Supplemental Table S13. Comparative numbers of the MADS‐box gene family
Supplemental Table S14. MADS‐box genes
Supplemental Table S15. Gene content of the chloroplast genome
Supplemental Table S16. Coding information of the mitochondrial genomes
Supplemental Table S17. Mitochondrial genome data

Supplementary material

Supplementary material

Supplementary material

Supplementary material

Supplementary material

Supplementary material

## Data Availability

The Whole Genome Shotgun project has been deposited at DNA Data Bank of Japan, European Nucleotide Archive, and GenBank under accession JAEPBF000000000. The genome version presented in this paper is JAEPBF010000000. *Passiflora organensis* genome annotations are available at https://genomevolution.org/coge/GenomeInfo.pl?gid=59125.
