# Supplementary material for: A genome sequence resource for the genus Passiflora, the genome of the wild diploid species Passiflora organensis
Source: Plant Genome. 2021 Jul 23;14(3):e20117. doi: 10.1002/tpg2.20117 (PMC12807398; doi:10.1002/tpg2.20117)
Supplement: Supplementary file 1 [file TPG2-14-e20117-s005.pdf]

A genome sequence resource for the genus *Passiflora*, the genome of the wild diploid species *Passiflora organensis*

Zirlane Portugal da Costa[1]; Alessandro de Mello Varani[2], Luiz Augusto Cauz-Santos[1,†]; Mariela Analía Sader[3]; Helena Augusto Giopatto[4]; Bruna Zirpoli[3]; Caroline Callot[5]; Stephane Cauet[5]; Willian Marande[5]; Jessica Luana Souza Cardoso[1]; Daniel Guariz Pinheiro[2]; João Paulo Kitajima[4]; Marcelo Carnier Dornelas[5]; Andrea Pedrosa-Harand[3]; Hélène Bergès[6]; Claudia Barros Monteiro-Vitorello[1]; Maria Lucia Carneiro Vieira[1*]

[1] Departamento de Genética, Escola Superior de Agricultura "Luiz de Queiroz", Universidade de São Paulo, 13418-900, Piracicaba, Brazil

[2] Departamento de Tecnologia, Faculdade de Ciências Agrárias e Veterinárias, Universidade Estadual Paulista, 14884-900, Jaboticabal, Brazil

[3] Departamento de Botânica, Universidade Federal de Pernambuco, 50670-901, Recife, Brazil

[4] Mendelics Análise Genômica AS, 04013-000, São Paulo, Brazil

[5] Departamento de Biologia Vegetal, Instituto de Biologia, Universidade Estadual de Campinas, 13083-862, Campinas, Brazil

[6] Institut National de la Recherche Agronomique (INRA), Centre National de Ressources Génomique Végétales (CNRGV), 31326, Castanet-Tolosan, France

[†] Present address: Department of Botany and Biodiversity Research, University of Vienna, 1030, Vienna, Austria

SUPPLEMENTAL FIGURES

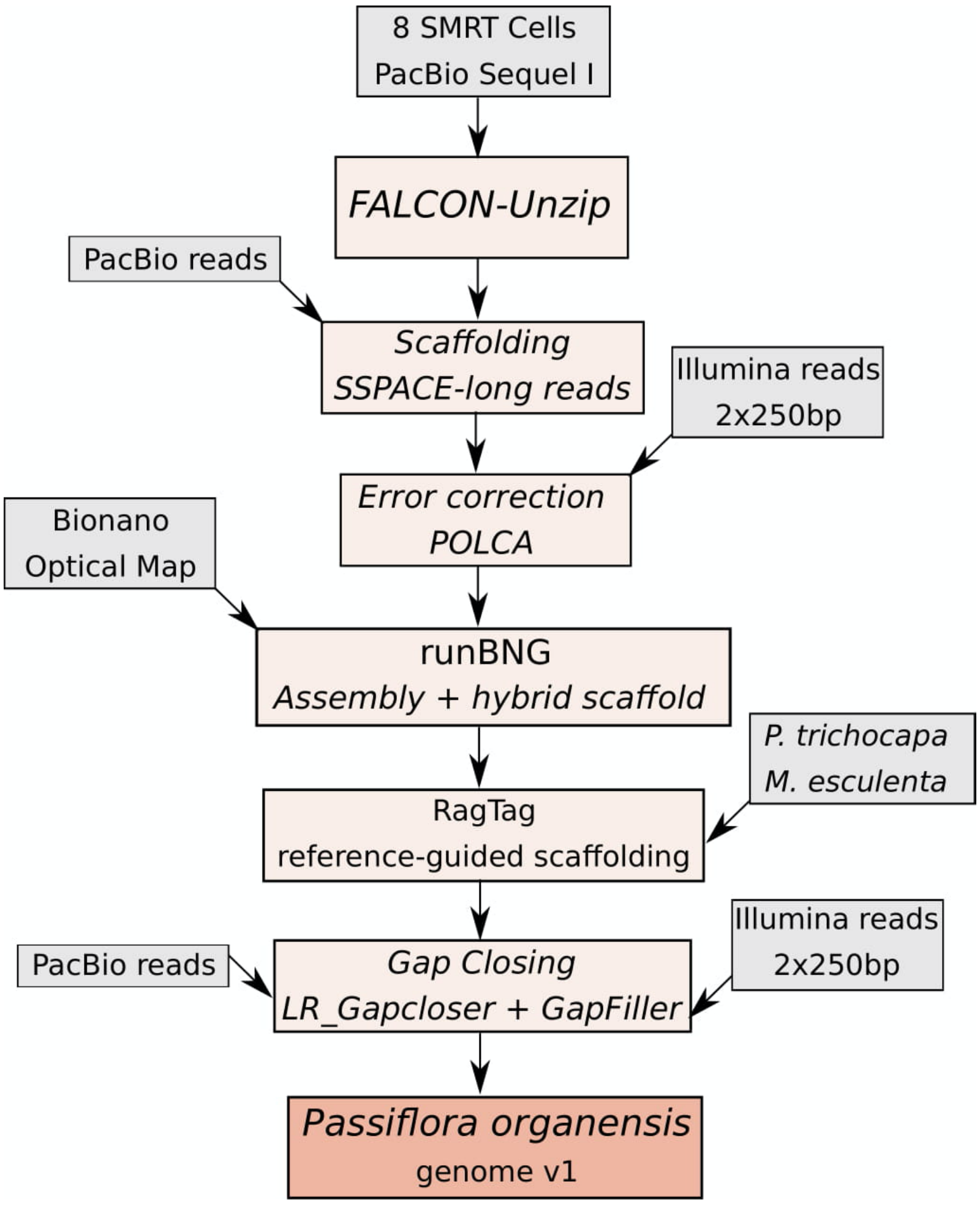

SUPPLEMENTAL FIGURE S1: Steps in the assembly of the *Passiflora organensis* genome. Eight PacBio Sequel II SMRT cells were assembled with Falcon-Unzip v. 1.8. 1 (Chin et al., 2016). The resulting assembly was further processed with SSPACE-LongRead v. 1.1 (Boetzer & Pirovano, 2014), using corrected and trimmed PacBio reads returned by CANU v. 2.1 (Koren et al., 2017) as input. Next, the assembly was processed and corrected with POLCA in MaSuRCA v. 3.4.2 (Zimin et al., 2017), using Illumina corrected reads with Trimmomatic v. 0.39 (Bolger et al., 2014) set to default parameters as input. The assembly was then used as a reference for Bionano molecule map *de novo* assembly using runBNG v. 2.0 (Yuan et al., 2017). The assembled Bionano molecules were used to produce Bionano hybrid scaffolding using the DLE1/BspTI enzyme. RaGOO v1.0.2 (Alonge et al., 2019) was also used to reference the genomes of *Populus trichocarpa* and *Mannhot esculenta* (parameters: minimum unique alignment length of 50 kb, alignment merge distance of 10 kb, and inferred gap sizes). Junctions were considered reliable only if the estimated gap length did not exceed 5 kb. Finally, two gap closing steps were implemented, the first using the PacBio reads corrected by LR_Gapcloser v.1.0 (Xu et al., 2018), and the second using the Illumina reads corrected by GapFiller v. 2.1.2 (Nadalin et al., 2012)

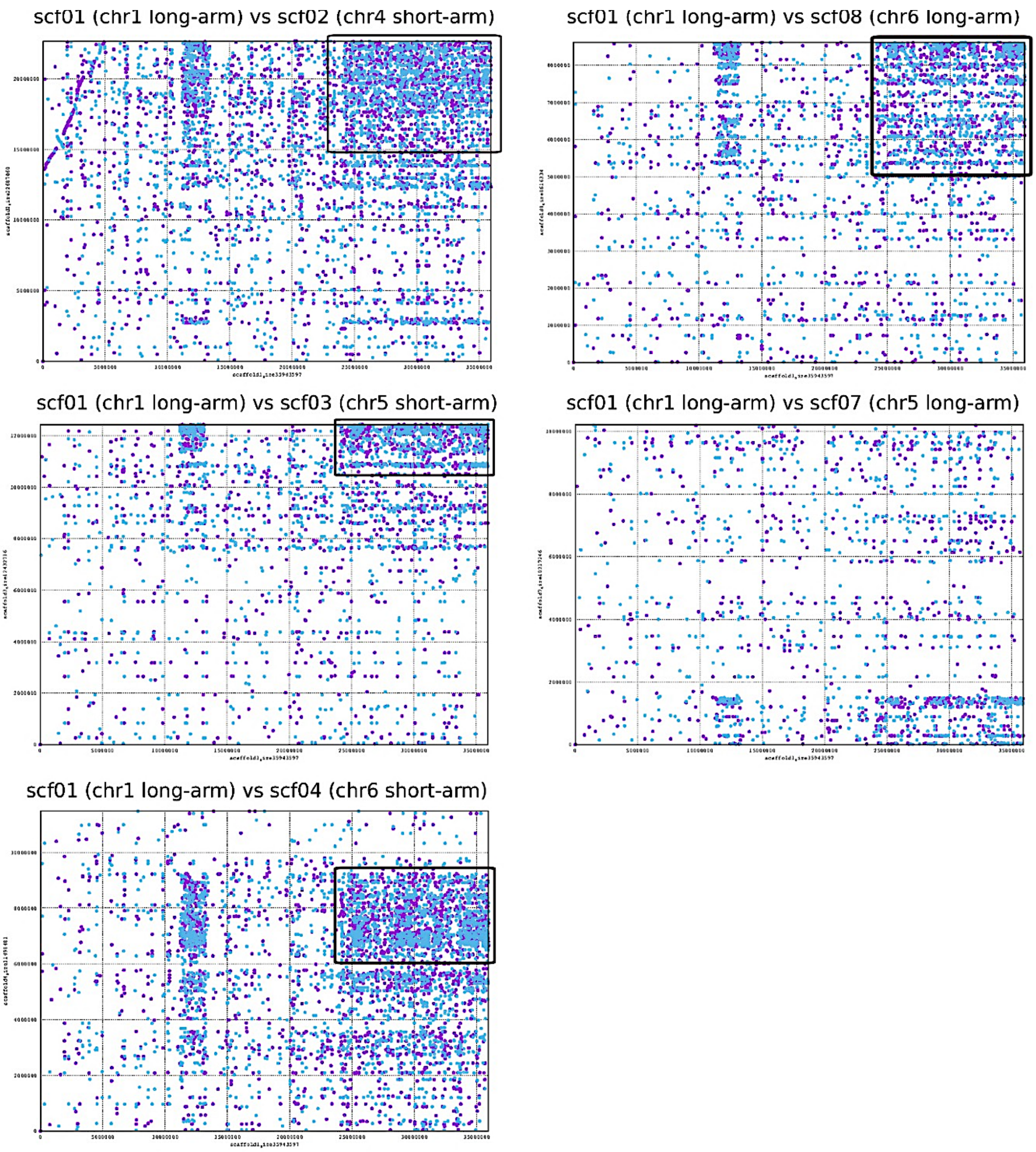

SUPPLEMENTAL FIGURE S2: MUMmer4 (Marçais et al., 2018) pairwise dot-plot alignment (default parameters) of scaffolds 1, 2, 3, 4, 7 and 8 representing assembled chromosome arms. Blue and purple dots indicate hits on each DNA strand. Black squares highlight putative pericentromeric and centromeric regions of strong sequence similarity. The regions in the black squares are confirmed TE-rich and gene-poor regions

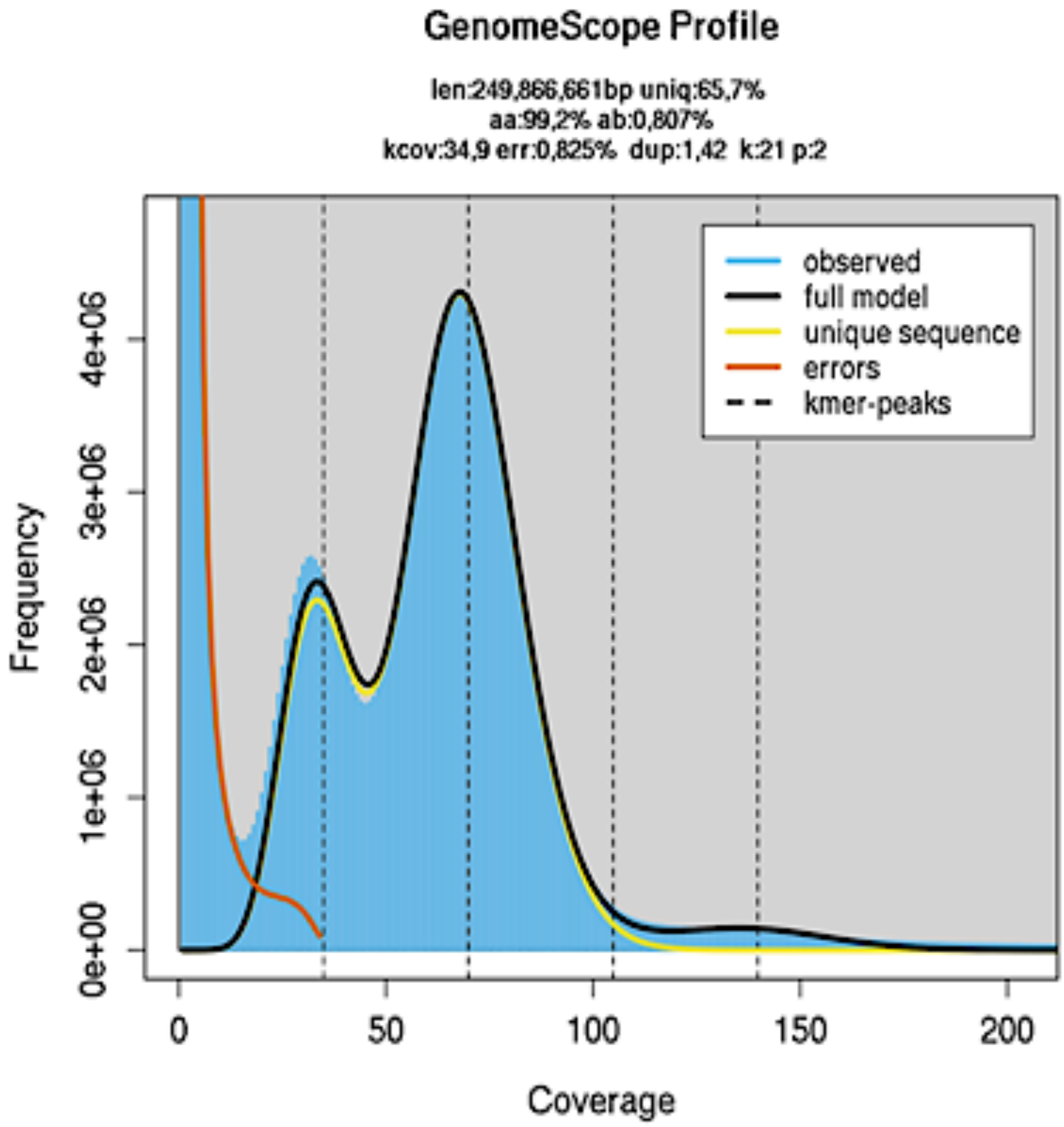

SUPPLEMENTAL FIGURE S3: GenomeScope k-mer profile plot of the *Passiflora organensis* genome dataset (based on Illumina sequencing runs that yielded 2×250 bp paired-end reads) showing the fit of the GenomeScope model (in black) for the observed k-mer frequencies (in blue). The peak pattern suggests a heterozygous diploid genome

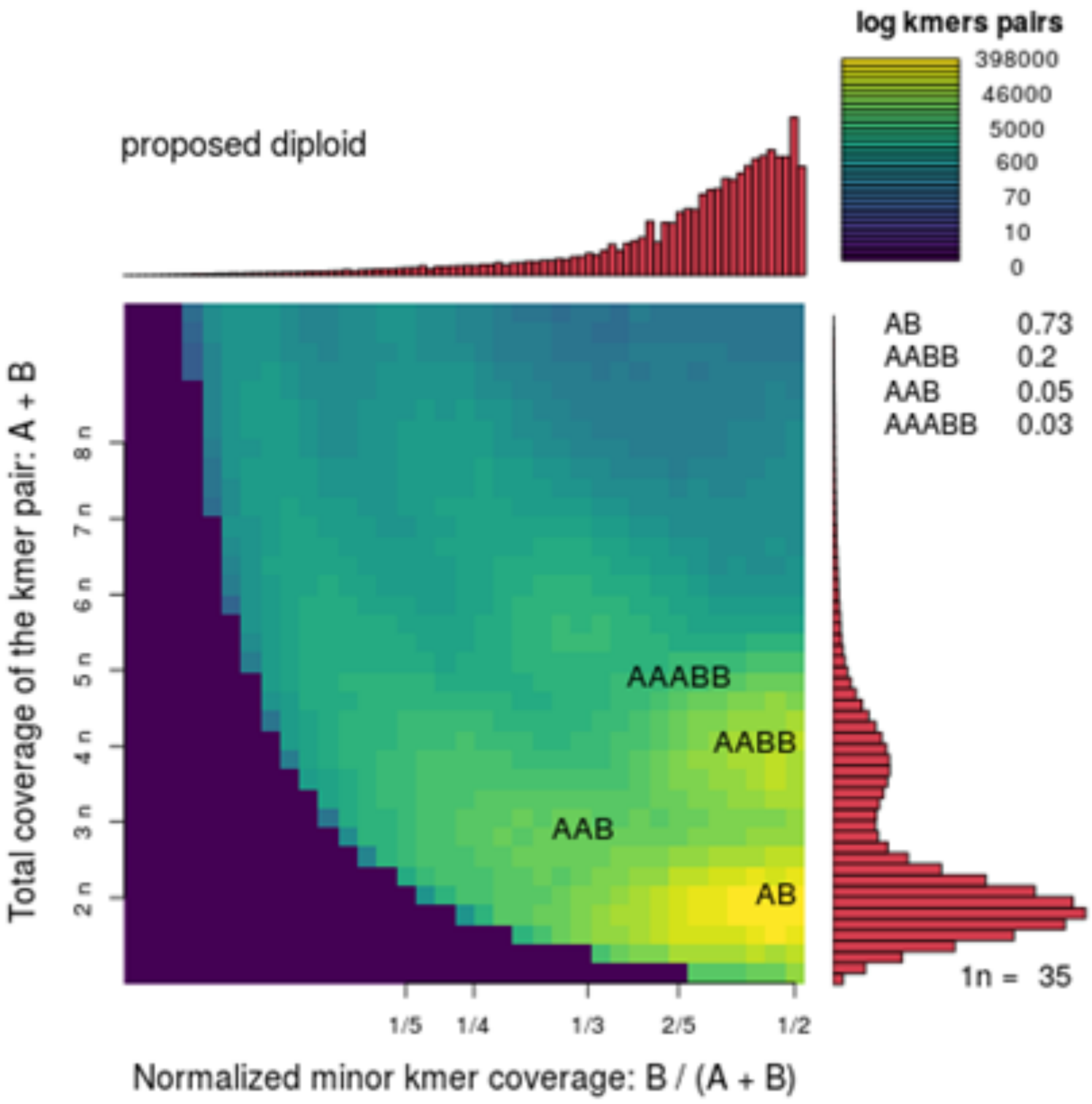

SUPPLEMENTAL FIGURE S4: Smudgeplot profile for the *Passiflora organensis* genome. The GenomeScope polyploidy model using 2× *p* negative binomial distributions strongly supports a diploid genome structure (AB 73%)

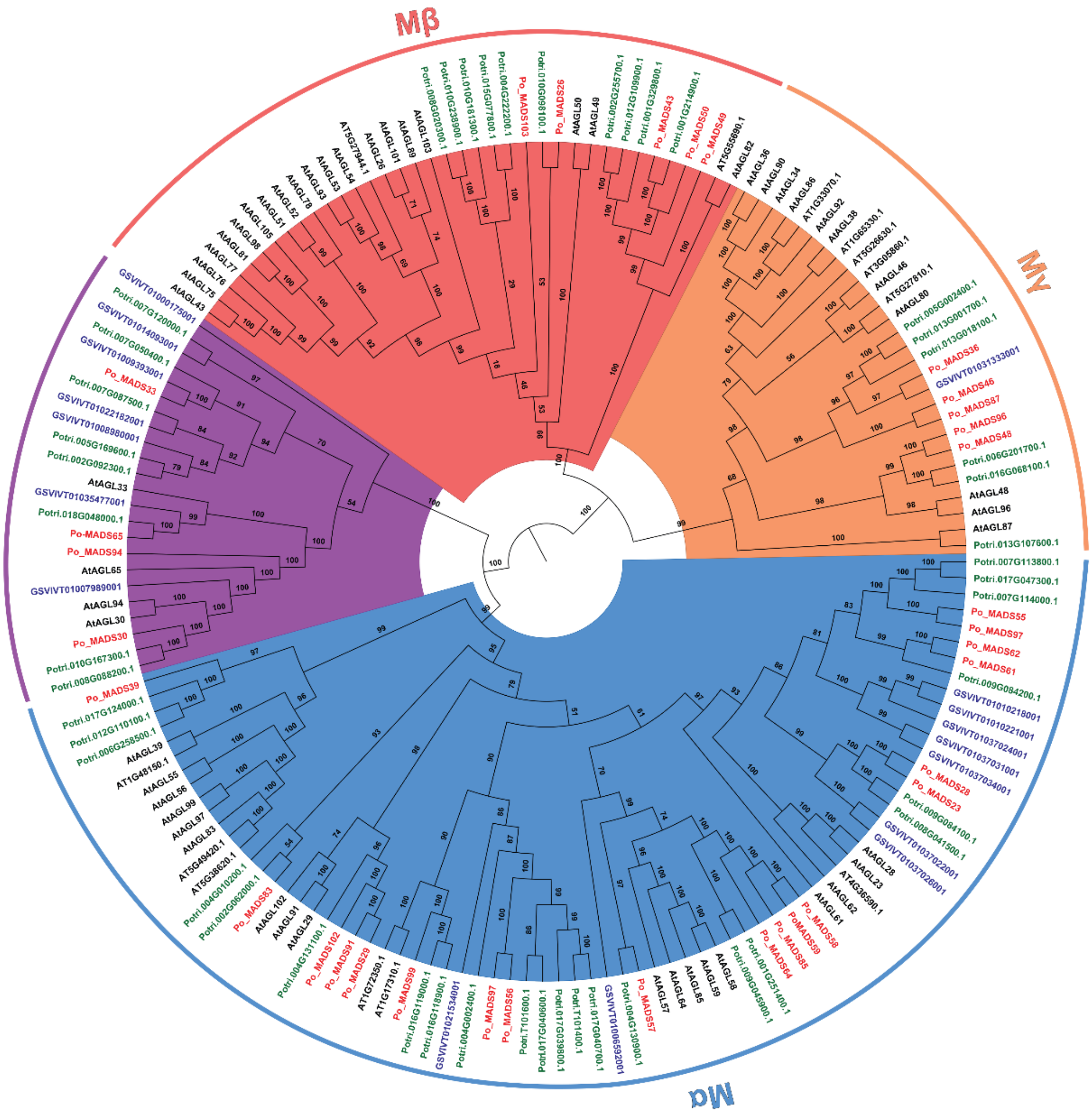

SUPPLEMENTAL FIGURE S5: Phylogenetic analysis of M-type MADS-box genes in *Passiflora organensis* (Po), *Arabidopsis thaliana* (At), *Populus trichocarpa* (Potri) and *Vitis vinifera* (GSVIV). The sequences were split into groups Mα, Mβ, Mγ and Mδ. The numbers at the nodes are bootstrap test results

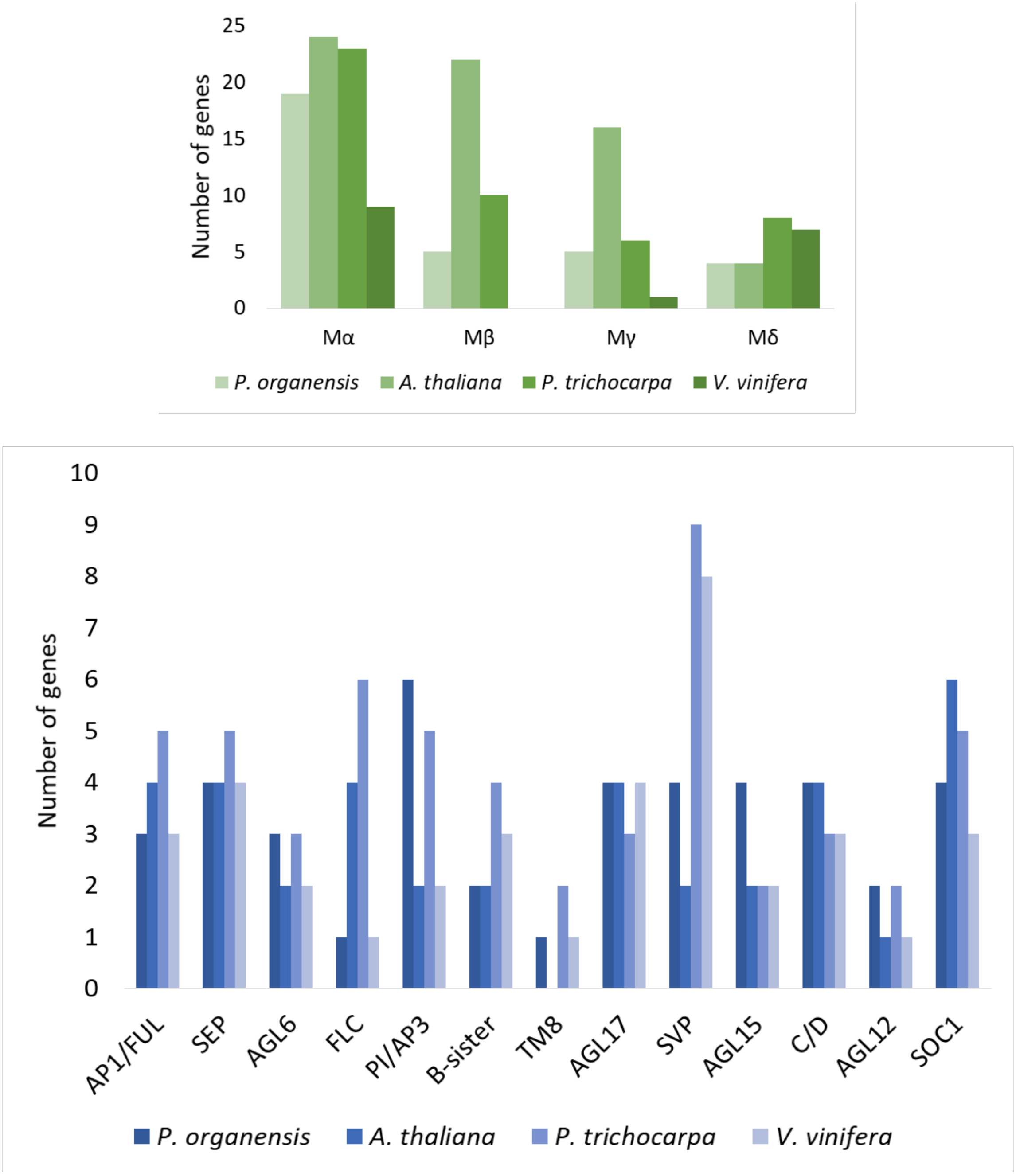

SUPPLEMENTAL FIGURE S6: Comparative numbers of M-type (above) and MIKC-type (below) members of the MADS-box gene family in *Passiflora organensis* and their counterparts in other plant species. The respective numbers for *Arabidopsis thaliana*, *Populus trichocarpa* and *Vitis vinifera* are taken from (Pařenicová et al., 2003; Leseberg et al., 2006; Grimplet et al., 2016)

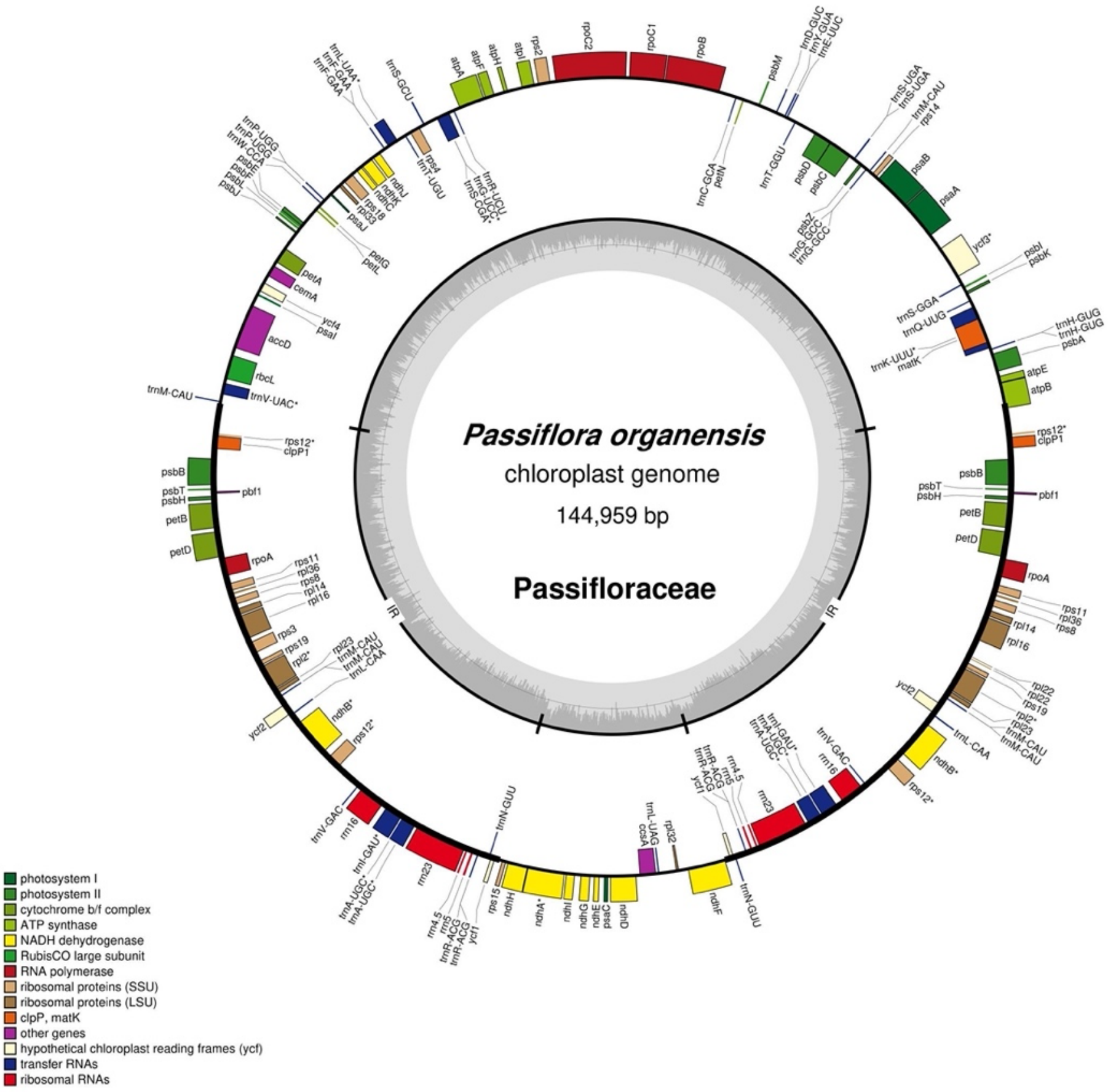

SUPPLEMENTAL FIGURE S7: Schematic circular representation of *Passiflora organensis* chloroplast DNA, indicating its main features and gene organization

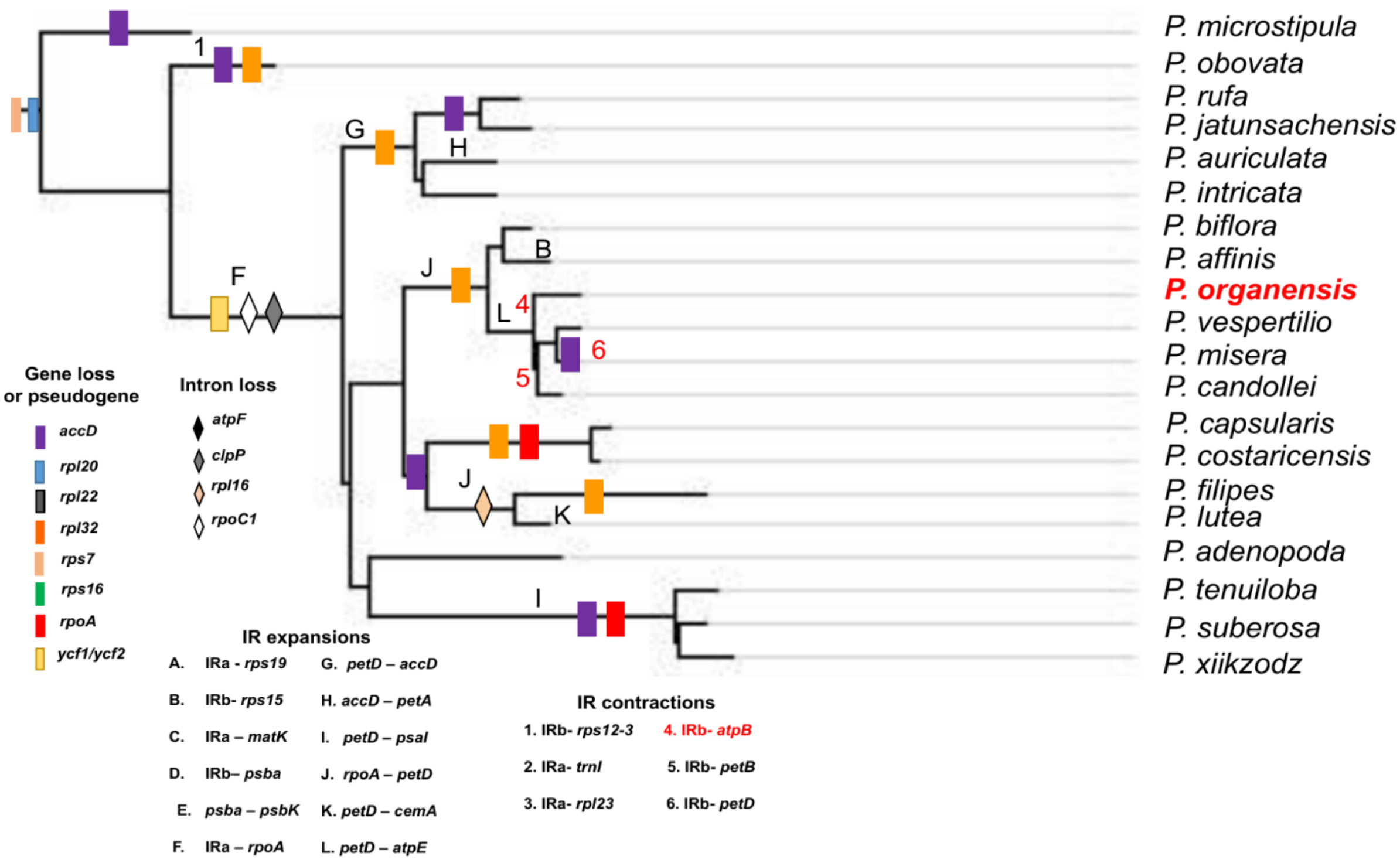

SUPPLEMENTAL FIGURE S8: Dendrogram showing *Passiflora* chloroplast genome rearrangements that occurred during the evolution of subgenus *Decaloba*. *Passiflora organensis* (highlighted in red) added to the analysis in the present study in accordance with Cauz-Santos et al. (2020)

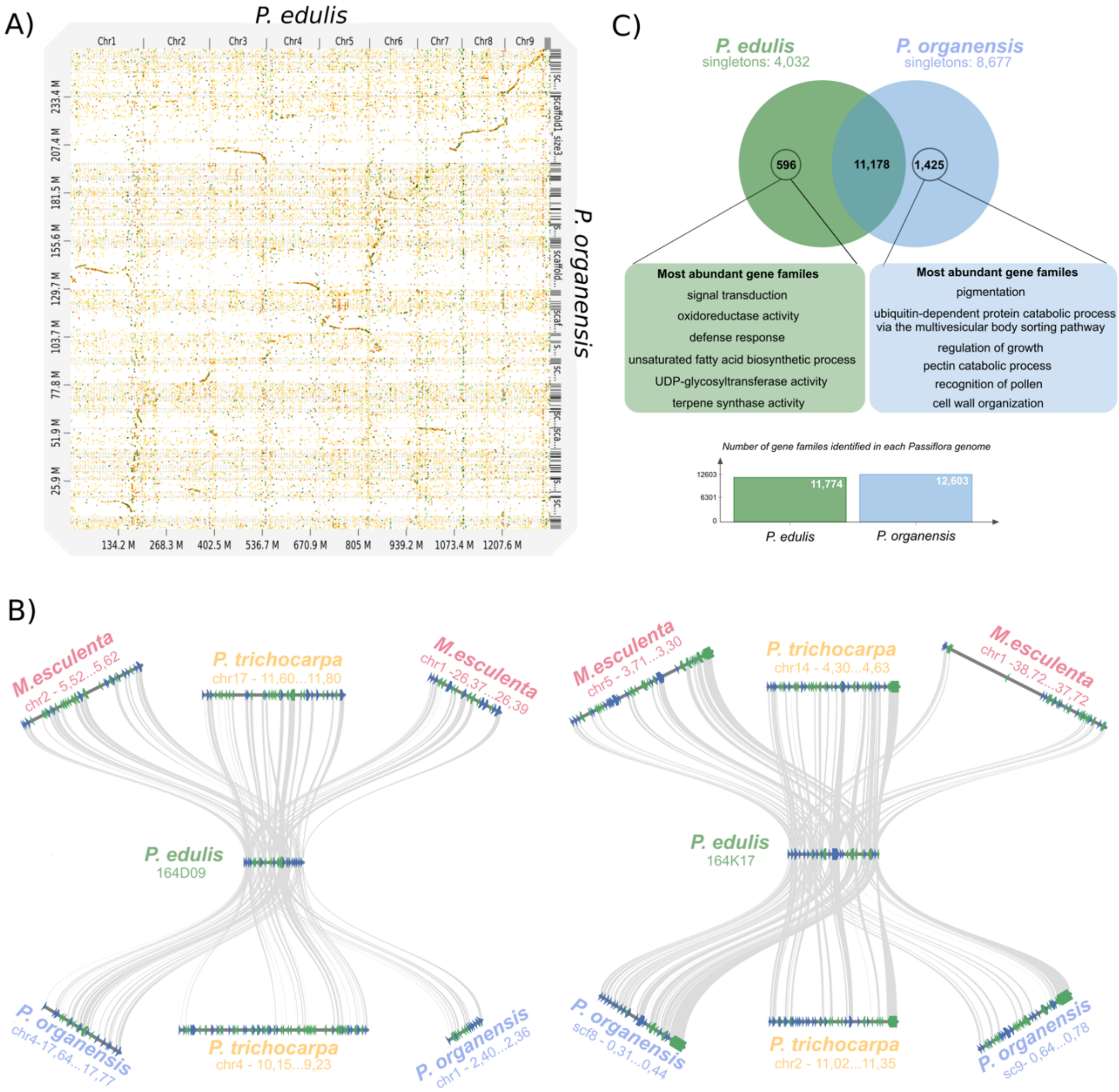

SUPPLEMENTAL FIGURE S9: Comparative genomic analysis of *Passiflora edulis* (Xia et al., 2021) and *Passiflora organensis*. **A** Pairwise dot-plot alignment (Cabanettes & Klopp, 2018) showing macroscale differences. **B** Microsyntenic segments shared by the genomes of *P. organensis*, *P. edulis*, *Manihot esculenta* and *Populus trichocarpa*. **C** Venn diagram showing the distribution of orthologous gene families in *P. edulis* and *P.* organensis. A total of 11,178 gene families were common to both species

SUPPLEMENTAL TABLES

SUPPLEMENTAL TABLE S1: Genome data (retrieved from Phytozome v. 13) used for reference-guided scaffolding of the assembly[a] and genome similarities among the Malpighiales species[b]. *Arabidopsis thaliana* was the outgroup

**Family**	**Species**	**Genome version**	**Reference**
Salicaceae	*Populus trichocarpa*[a,b]	Ptrichocarpa_533_v4.1	(Tuskan et al., 2006)
Salicaceae	*Salix purpurea*[b]	Spurpurea_519_v5.1	(Smart et al.)
Euphorbiaceae	*Manihot esculenta*[a,b]	Mesculenta_520_v7.1	(Prochnik et al., 2012)
Euphorbiacea	*Ricinus communis*[b]	Rcomunis_119_v0.1	(Chan et al., 2010)
Brassicaceae	*Arabidopsis thaliana*[b]	Athaliana_167_TAIR10	(Lamesch et al., 2012)

SUPPLEMENTAL TABLE S2: Complementary statistics for *Passiflora organensis* genome sequencing data

**Raw data**						
	**Library**	**Insert size (bp)**	**Read length (bp)**	**Read number**	**Sequence coverage**	**Total (Gb)**
DNA_SEQ	Pacbio_1	-	2,556,547,244	365,246	10	33
	Pacbio_2	-	2,877,138,304	425,860	11	
	Pacbio_3	-	2,244,653,506	317,935	9	
	Pacbio_4	-	2,121,946,871	306,297	8	
	Pacbio_5	-	2,410,804,419	354,338	9	
	Pacbio_6	-	1,947,104,022	289,348	7	
	Pacbio_7	-	1,947,573,410	284,154	7	
	Pacbio_8	-	1,031,171,040	159,801	4	
	Illumina (2×250 bp)	450	250	56,183,685	112	60
RNA_SEQ (2×100 bp)	Illumina_1	350	100	40,588,361	32	30
	Illumina_2	350	100	35,943,699	29	
	Illumina_3	350	100	37,740,452	28	

SUPPLEMENTAL TABLE S3: Statistics for *Passiflora organensis* assembly and annotation

Type	Parameter	Value
**Assembly**	Total assembly size (Mbp)	259,301,974
	Number of scaffolds	360
	Total scaffold length as percentage of known genome size	123.5%
	Longest scaffold	35,942,160
	Shortest scaffold	4,400
	Number of scaffolds > 500 nt	360 (100.0%)
	Number of scaffolds > 1K nt	360 (100.0%)
	Number of scaffolds > 10K nt	345 (95.8%)
	Number of scaffolds > 100K nt	189 (52.5%)
	Number of scaffolds > 1M nt	38 (10.6%)
	Mean scaffold size	720,283
	Median scaffold size	111,749
	N50 scaffold length	8,258,683
	L50 scaffold count	9
	NG50 scaffold length	10,217,281
	LG50 scaffold count	7
	N50 scaffold - NG50 scaffold length difference	1,958,598
	Scaffold %A	30.84
	Scaffold %C	19.15
	Scaffold %G	19.15
	Scaffold %T	30.84
	Scaffold %N	0.04
	Scaffold N nt	96,812
**Annotation**	Number of genes	25,327
	Number of CDS	40,870
	Mean gene length	3,200
	Annotated genes	24,000
	Number of genes with GO terms assigned	21,019
	Genome covered with genes	31.3 %
	Genome covered with TEs	58.55 %
	Number of mRNAs	40,870
	Number of exons	261,761
	Number of introns	220,891
	Overlapping genes	2,154
	Contained genes	558
	CDS: complete	40,818
	CDS: start, no stop	14
	CDS: stop, no start	25

Type	Parameter	Value
	CDS: no stop, no start	13
	Total gene length	81,057,801
	Total mRNA length	150,138,272
	Total exon length	79,472,490
	Total intron length	71,107,564
	Total CDS length	51,116,295
	Shortest gene	177
	Shortest mRNA	177
	Shortest exon	3
	Shortest intron	20
	Shortest CDS	141
	Longest gene	199,873
	Longest mRNA	199,873
	Longest exon	7,872
	Longest intron	198,165
	Longest CDS	16,230
	Mean gene length	3,200
	Mean intron length	322
	Mean CDS length	1,251
	Mean mRNAs per gene	2
	Mean exons per mRNA	6
	Mean introns per mRNA	5

SUPPLEMENTAL TABLE S4: *Passiflora organensis* raw Bionano molecule map status based on the PacBio assembled genome

Type	Value
Number of molecules	8,945,541
Total length of molecules	588,853 Mbp
N50 Length	117 Kbp
Minimum length of molecules	15 Kbp
Maximum length of molecules	2,801.25 Kbp
Average length of molecules	65.8264 Kbp
Length standard deviation	95.5113 Kbp
Total number of labels	101,330,153
Label density (/100Kb)	17.2081
Maximum signal/noise ratio (SNR)	7,516.73
Minimum signal/noise ratio (SNR)	2.27

Type	Value
Average signal/noise ratio (SNR)	155.869
Maximum avgIntensity	103,296.00
Minimum avgIntensity	62.15
Average avgIntensity	2,537.83

SUPPLEMENTAL TABLE S5: *Passiflora organensis* Bionano assembled molecule map status

Type	Value
Count	137
Minimal length	0.360
Median length	1.768
Mean length	3.204
N50 length	5.679
Max length	33.326
Total length	438.921
Label density (/100Kb)	16,000

Lengths expressed in Mbp

SUPPLEMENTAL TABLE S6: Scaffold number and length showing assembled telomeric repeats at one end, mapped to chromosome arm information

Scaffold			
**Number**	**Length (bp)**	**Assignment**	**Evidence (*)**
1	35,942,160	Long arm of chromosome 1	Kar, Bioinfo
2	22,685,330	Short arm of chromosome 4	Kar, Bioinfo and Sat
3	12,431,908	Long arm of chromosome 5	Kar, Bioinfo
4	11,497,491	Short arm of chromosome 6	Kar, Bioinfo
7	10,217,281	Short arm of chromosome 5	Kar, Bioinfo and Sat
8	8,616,044	Long arm of chromosome 6	Kar, Bioinfo
71	556,355	Short arm of chromosome 3	Sat, Bioinfo
92	390,292	Long arm of chromosome 2	Sat, Bioinfo
**Total**	102,336,861		

(*) Based on the *Passiflora organensis* karyotype (Kar); FISH hybridization of Satellite DNAs (Sat); Scaffold sizes and pericentromeric/centromeric regions identified by the dot-plot approach (Bioinfo details are given in Supplemental Figure S2)

SUPPLEMENTAL TABLE S7: Completeness evaluation of *Passiflora organensis* genome assembly using BUSCO v. 4.1.4 (Seppey et al., 2019) with the embryophyta_odb10 database

**Type**	**Number**	**Percentage**
Complete BUSCOs (C)	1,587	98.4
Complete and single-copy BUSCOs (S)	1,528	94.7
Complete and duplicated BUSCOs (D)	59	3.7
Fragmented BUSCOs (F)	7	0.4
Missing BUSCOs (M)	20	1.2
Total BUSCO groups searched	1,614	100

SUPPLEMENTAL TABLE S9: Transposable elements identified in the *Passiflora organensis* genome

**Element classification**	**Number of elements**	**Total occurrences**	**Length occupied (bp)**	**Percentage of genome**
**Retroelements**	**2,383**	**87,946**	**103,381,492**	**39.87**
SINEs	51	1,205	223,797	0.09
LINEs	550	10,501	14,927,780	5.76
**LTR elements**	**1,654**	**74,487**	**87,659,639**	**33.81**
LTR-LARD	55	2,772	1,917,396	0.74
LTR-TRIM	102	4,217	2,002,021	0.77
**LTR/Copia**	**257**	**10,608**	**10,219,947**	**3.94**
Copia|Ale	30	891	898,763	0.35
Copia|Alesia	2	27	46,626	0.02
Copia|Angela	4	72	73,282	0.03
Copia|Bianca	27	1,625	2,524,762	0.97
Copia|Ivana	61	1,555	1,601,743	0.62
Copia|Ikeros	15	1,628	802,567	0.31
Copia|TAR	3	177	300,669	0.12
Copia|Tork	40	1,648	2,028,187	0.78
Copia|Unknown	75	2,985	1,943,348	0.75
**LTR/Gypsy**	**1,235**	**56,182**	**73,332,188**	**28.28**
**Chromovirus**	**900**	**40,633**	**60,878,526**	**23.48**
Reina	70	1,593	2,023,405	0.78

Tekay	760	35,271	54,498,503	21.02
Galadriel	33	2,587	3,534,222	1.36
Others/Unclassified	37	1,182	822,396	0.32
**Non-Chromovirus**	**61**	**6,662**	**5,909,714**	**2.28**
OTA|Athila	61	6,662	5,909,714	2.28
Gypsy|Unknown	274	8,887	6,543,948	2.52
**DIRS**	**4**	**46**	**37,512**	**0.01**
**LTR Unknown**	**5**	**708**	**188,087**	**0.07**
**Pararetrovirus**	**48**	**598**	**586,815**	**0.23**
**RNA/Unknown**	**76**	**1,753**	**570,276**	**0.22**
**DNA transposons**	**254**	**8,774**	**7,225,604**	**2.79**
Maverick	2	42	20,880	0.01
MITE	44	889	343,102	0.13
Helitron	97	2,044	1,987,483	0.77
TIR	60	4,328	3,309,234	1.28
hAT	26	574	660,344	0.25
MuDR/Mutator	8	252	209,892	0.08
PIF/Harbinger	8	299	233,818	0.09
EnSpm/CACTA	7	316	443,038	0.17
Others	2	30	17,813	0.01
**Unclassified**	**2,945**	**71,992**	**41,027,637**	**15.82**
**Interspersed repeats**	**5,582**	**168,712**	**151,634,733**	**58.48**
**SSRs**	**1,156**	**2,645**	**182,730**	**0.07**
**TOTAL**	**6,738**	**171,357**	**151,817,463**	**58.55**

SUPPLEMENTARY TABLE S13: Comparative numbers of members of M-type and MIKC-type MADS-box genes in *Passiflora organensis* and their counterparts in other plant species. The respective numbers for *Arabidopsis thaliana, Populus trichocarpa* and *Vitis vinifera* were obtained from Pařenicová et al. (2003); Leseberg et al. (2006) and Grimplet et al. (2016)

**M-type**	***P. organensis***	***A. thaliana***	***P. trichocarpa***	***V. vinifera***
Mα	19	24	23	9
Mβ	5	22	10	-
Mγ	5	16	6	1
Mδ	4	4	8	7
**MIKC**	***P. organensis***	***A. thaliana***	***P. trichocarpa***	***V. vinifera***
AP1/FUL	3	4	5	3
SEP	4	4	5	4
AGL6	3	2	3	2

FLC	1	4	6	1
PI/AP3	6	2	5	2
B-sister	2	2	4	3
TM8	1	-	2	1
AGL17	4	4	3	4
SVP	4	2	9	8
AGL15	4	2	2	2
C/D	4	4	3	3
AGL12	2	1	2	1
SOC1	4	6	5	3

SUPPLEMENTAL TABLE S15: Gene content of the *Passiflora organensis* chloroplast genome

**Category**	**Genes**
Subunits of Photosystem I	*psaA*, *psaB*, *psaC*, *psaI*, *psaJ*
Subunits of Photosystem II	*psbA*, *psbB*[b], *psbC*, *psbD*, *psbE*, *psbF*, *psbH*[b] , *psbI*, *psbJ*, *psbK*, *psbL*, *psbM*, *psbN*[b] , *psbT*[b] , *psbZ*
Subunits of cytochrome b/f complex	*petA*, *petB*[a,b], *petD*[a,b], *petG*, *petL*, *petN*
Subunits of ATP synthase	*atpA*, *atpB*, *atpE*, *atpF*, *atpH*, *atpI*
Large subunit of Rubisco	*rbcL*
Subunits of NADH-dehydrogenase	*ndhA*[a], *ndhB*[a,b], *ndhC*, *ndhD*, *ndhE*, *ndhF*, *ndhG*, *ndhH*, *ndhI*, *ndhJ*, *ndhK*
Proteins of large ribosomal subunit	*rpl2*[a,b], *rpl14*[b], *rpl16* [a,b], *rpl23*[b], *rpl33*, *rpl36*[b]
Proteins of small ribosomal subunit	*rps2*, *rps3*[b], *rps4*, *rps8*[b], *rps11*[b], *rps12*[a,b,c], *rps14*, rps15, *rps18*, *rps19*[b]
Subunits of RNA polymerase	*rpoA*[b], *rpoB*(Yang & Wang, 2020), *rpoC1*, *rpoC2*
Cytochrome c biogenesis	*ccsA*
Acetyl coA carboxylase	*accD*
Maturase	*matk*
Protease	*clpP* [b]
Envelope membrane protein	*cemA*
Conserved hypothetical genes	*ycf3*[a], *ycf4*
Ribosomal RNAs	*rrn4*.5[b], *rrn5*[b], *rrn16*[b], *rrn23*[b]
Pseudogene	*ycf1*[b], *ycf2*[b], *rpl32*
Transfer RNAs	*trnA-UGC*[a,b], *trnC-GCA*, *trnD-GUC*, *trnE-UUC*, *trnF-GAA*, *trnfM-CAU*, *trnG-UCC*[a], *trnG-GCC*, *trnH-GUG*, *trnI-CAU*[b], *trnI-GAU*[a,b], *trnK-UUU* [a], *trnL-CAA*[b], *trnL-UAA* [a], *trnL-UAG*, *trnM-CAU*, *trnN-GUU*[b], *trnP-UGG*, *trnQ-UUG*, *trnR-ACG*[b], *trnR-UCU*, *trnS-GCU*, *trnS-GGA*, *trnS-UGA*, *trnT-GGU*, *trnT-UGU*, *trnV-GAC*[b], *trnV-UAC*[a], *trnW-CCA*, *trnY-GUA*

[a]Intron-containing gene; [b]Two gene copies in the IRs; [c]Gene divided into two independent transcription units

SUPPLEMENTAL TABLE S16: Coding information (number of amino acids for each protein sequence) in the mitochondrial genomes of *Passiflora organensis, P. edulis* and *Arabidopsis thaliana*

**Category**	**Gene**	**Genome**		
		P. organensis	***P. edulis*(Yang & Wang, 2020)**	***A. thaliana*(Marienfeld et al., 1999)**
Complex I	*nad1*	330 (3)[a]	295 (3)[a]	324 (4)[a]
	nad2	488 (4)[a]	488 (4)[a]	307 (4)[a]
	nad3	118	118	118
	nad4	495 (3)	495 (3)	494 (3)
	nad4L	100	100	100
	nad5	665 (3)[a]	663 (3)[a]	668 (4)[a]
	nad6	204	204/204	206
	nad7	394 (4)	394; 394(4)	394 (4)
	nad9	190	190	191
Complex II	*sdh2*	--	--	--
	sdh3	95	95	--
Complex III	*cob*	392	390	393
Complex IV	*cox1*	527	608	527
	cox2	258	258; 258	260 (1)
	cox3	265	265	265
Complex V	*atp1*	503	509	507
	atp4	198	198	--
	atp6	391	385	385; 349
	atp8	162	162	158
	atp9	103	83	85
Cytochrome-c biogenesis	*ccmB*	206	206; 206	206
	ccmC	250	250	256
	ccmFC	436 (1)	436 (1)	543 (1)
	ccmFN1	581	582	382
	ccmFN2	--	--	203
Protein transport	*mttB*	307	283	286
Other ORFs	*matR*	657	656	--
Ribosomal proteins	*rps1*	313	222 (1)	--
	rps3	558	562 (1)	556 (1)
	rps4	388	317/317	362
	rps7	--	--	148
	rps12	123; 123	125	125

rps19	94[b]	94	--
rpl5	182	184	185
rpl16	158	82	--
rpl10	154 [b]	154	179

[a] includes trans-splicing introns; ψ pseudogenes or misassembled regions; gene copy numbers are in parenthesis; [b]located in the circular mtDNA molecule

REFERENCES

Alonge, M., S. Soyk, S. Ramakrishnan, X. Wang, S. Goodwin, F.J. Sedlazeck, Z.B. Lippman, & M.C. Schatz. 2019. RaGOO: fast and accurate reference-guided scaffolding of draft genomes. *Genome Biology*, 20:224. doi:10.1186/s13059-019-1829-6

Boetzer, M., & W. Pirovano. 2014. SSPACE-LongRead: scaffolding bacterial draft genomes using long read sequence information. *BMC Bioinformatics*, 15:211. doi:10.1186/1471-2105-15-211

Bolger, A.M., M. Lohse, & B. Usadel. 2014. Trimmomatic: a flexible trimmer for Illumina sequence data. *Bioinformatics*, 30:2114–2120. doi:10.1093/bioinformatics/btu170

Cabanettes, F., & C. Klopp. 2018. D-GENIES: Dot plot large genomes in an interactive, efficient and simple way. *PeerJ*, 2018:e4958. doi:10.7717/peerj.4958

Cauz-Santos, L.A., Z.P. Costa, C. Callot, S. Cauet, M.I. Zucchi, H. Bergès, C. van den Berg, & M.L.C. Vieira. 2020. A repertory of rearrangements and the loss of an inverted repeat region in Passiflora chloroplast genomes. *Genome Biology and Evolution*, 12:1841–1857. doi:10.1093/gbe/evaa155

Chan, A.P., J. Crabtree, Q. Zhao, H. Lorenzi, J. Orvis, D. Puiu, A. Melake-Berhan, K.M. Jones, J. Redman, G. Chen, E.B. Cahoon, M. Gedil, M. Stanke, B.J. Haas, J.R. Wortman, C.M. Fraser-Liggett, J. Ravel, & P.D. Rabinowicz. 2010. Draft genome sequence of the oilseed species *Ricinus communis*. *Nature Biotechnology*, 28:951–956. doi:10.1038/nbt.1674

Chin, C.S., P. Peluso, F.J. Sedlazeck, M. Nattestad, G.T. Concepcion, A. Clum, C. Dunn, R. O'Malley, R. Figueroa-Balderas, A. Morales-Cruz, G.R. Cramer, M.

Delledonne, C. Luo, J.R. Ecker, D. Cantu, D.R. Rank, & M.C. Schatz. 2016. Phased diploid genome assembly with single-molecule real-time sequencing. *Nature Methods*, 13:1050–1054. doi:10.1038/nmeth.4035

Grimplet, J., J.M. Martínez-Zapater, & M.J. Carmona. 2016. Structural and functional annotation of the MADS-box transcription factor family in grapevine. *BMC Genomics*, 17:80. doi:10.1186/s12864-016-2398-7

Koren, S., B.P. Walenz, K. Berlin, J.R. Miller, N.H. Bergman, & A.M. Phillippy. 2017. Canu: scalable and accurate long-read assembly via adaptive k-mer weighting and repeat separation. *Genome Research*, 27:722–736. doi:10.1101/gr.215087.116

Lamesch, P., T.Z. Berardini, D. Li, D. Swarbreck, C. Wilks, R. Sasidharan, R. Muller, K. Dreher, D.L. Alexander, M. Garcia-Hernandez, A.S. Karthikeyan, C.H. Lee, W.D. Nelson, L. Ploetz, S. Singh, A. Wensel, & E. Huala. 2012. The *Arabidopsis* Information Resource (TAIR): improved gene annotation and new tools. *Nucleic Acids Research*, 40:D1202-10. doi:10.1093/nar/gkr1090

Leseberg, C.H., A. Li, H. Kang, M. Duvall, & L. Mao. 2006. Genome-wide analysis of the MADS-box gene family in *Populus trichocarpa*. *Gene*, 378:84–94. doi:10.1016/j.gene.2006.05.022

Marçais, G., A.L. Delcher, A.M. Phillippy, R. Coston, S.L. Salzberg, & A. Zimin. 2018. MUMmer4: a fast and versatile genome alignment system. *PLOS Computational Biology*, 14:e1005944. doi:10.1371/journal.pcbi.1005944

Marienfeld, J., M. Unseld, & A. Brennicke. 1999. The mitochondrial genome of *Arabidopsis* is composed of both native and immigrant information. *Trends in Plant Science*, 4:495–502. doi:10.1016/S1360-1385(99)01502-2

Nadalin, F., F. Vezzi, & A. Policriti. 2012. GapFiller: a de novo assembly approach to fill the gap within paired reads. *BMC Bioinformatics*, 13:S8. doi:10.1186/1471-2105-13-S14-S8

Pařenicová, L., S. De Folter, M. Kieffer, D.S. Horner, C. Favalli, J. Busscher, H.E. Cook, R.M. Ingram, M.M. Kater, B. Davies, G.C. Angenent, & L. Colombo. 2003. Molecular and phylogenetic analyses of the complete MADS-Box transcription factor family in *Arabidopsis*: new openings to the MADS world. *Plant Cell*,

15:1538–1551. doi:10.1105/tpc.011544

Prochnik, S., P.R. Marri, B. Desany, P.D. Rabinowicz, C. Kodira, M. Mohiuddin, F. Rodriguez, C. Fauquet, J. Tohme, T. Harkins, D.S. Rokhsar, & S. Rounsley. 2012. The cassava genome: current progress, future directions. *Tropical Plant Biology*, 5:88–94. doi:10.1007/s12042-011-9088-z

Seppey, M., M. Manni, & E.M. Zdobnov. 2019. BUSCO: assessing genome assembly and annotation completeness. *Methods in Molecular Biology*, 1962:227–245. doi:10.1007/978-1-4939-9173-0_14

Smart, L., C. Town, S. DiFazio, G. Tuskan, & J. Schmutz. *Salix Purpurea* v1.0. https://phytozome.jgi.doe.gov/pz/portal.html#!info?alias=Org_Spurpurea (accessed December 13, 2020).

Tuskan, G.A., S. DiFazio, S. Jansson, J. Bohlmann, I. Grigoriev, U. Hellsten, N. Putnam, S. Ralph, S. Rombauts, A. Salamov, J. Schein, L. Sterck, A. Aerts, R.R. Bhalerao, R.P. Bhalerao, D. Blaudez, W. Boerjan, A. Brun, A. Brunner, V. Busov, M. Campbell, J. Carlson, M. Chalot, J. Chapman, G.-L. Chen, D. Cooper, P.M. Coutinho, J. Couturier, S. Covert, Q. Cronk, R. Cunningham, J. Davis, S. Degroeve, A. Dejardin, C. DePamphilis, J. Detter, B. Dirks, I. Dubchak, S. Duplessis, J. Ehlting, B. Ellis, K. Gendler, D. Goodstein, M. Gribskov, J. Grimwood, A. Groover, L. Gunter, B. Hamberger, B. Heinze, Y. Helariutta, B. Henrissat, D. Holligan, R. Holt, W. Huang, N. Islam-Faridi, S. Jones, M. Jones-Rhoades, R. Jorgensen, C. Joshi, J. Kangasjarvi, J. Karlsson, C. Kelleher, R. Kirkpatrick, M. Kirst, A. Kohler, U. Kalluri, F. Larimer, J. Leebens-Mack, J.-C. Leple, P. Locascio, Y. Lou, S. Lucas, F. Martin, B. Montanini, C. Napoli, D.R. Nelson, C. Nelson, K. Nieminen, O. Nilsson, V. Pereda, G. Peter, R. Philippe, G. Pilate, A. Poliakov, J. Razumovskaya, P. Richardson, C. Rinaldi, K. Ritland, P. Rouze, D. Ryaboy, J. Schmutz, J. Schrader, B. Segerman, H. Shin, A. Siddiqui, F. Sterky, A. Terry, C.-J. Tsai, E. Uberbacher, P. Unneberg, J. Vahala, K. Wall, S. Wessler, G. Yang, T. Yin, C. Douglas, M. Marra, G. Sandberg, Y. Van de Peer, & D. Rokhsar. 2006. The genome of black cottonwood, *Populus trichocarpa* (Torr. & Gray). *Science*, 313:1596–1604. doi:10.1126/science.1128691

Xia, Z., D. Huang, S. Zhang, W. Wang, F. Ma, B. Wu, Y. Xu, B. Xu, D. Chen, M. Zou, H. Xu, X. Zhou, R. Zhan, & S. Song. 2021. Chromosome-scale genome assembly

provides insights into the evolution and flavor synthesis of passion fruit (*Passiflora edulis* Sims). *Horticulture Research*, 8:14. doi:10.1038/s41438-020-00455-1

Xu, G.C., T.J. Xu, R. Zhu, Y. Zhang, S.Q. Li, H.W. Wang, & J.T. Li. 2018. LR-Gapcloser: a tiling path-based gap closer that uses long reads to complete genome assembly. *GigaScience*, 8:1–14. doi:10.1093/gigascience/giy157

Yang, J., & X.-A. Wang. 2020. The complete mitochondrial genome of a yellow passion fruit (*Passiflora edulis* Sims. f. flavicarpa Deg.) in China and phylogenetic relationships. *Mitochondrial DNA Part B*, 5:1598–1600. doi:10.1080/23802359.2020.1742622

Yuan, Y., P.E. Bayer, H.-T. Lee, & D. Edwards. 2017. runBNG: a software package for BioNano genomic analysis on the command line. *Bioinformatics*, 33:3107–3109. doi:10.1093/bioinformatics/btx366

Zimin, A. V., D. Puiu, M.C. Luo, T. Zhu, S. Koren, G. Marçais, J.A. Yorke, J. Dvořák, & S.L. Salzberg. 2017. Hybrid assembly of the large and highly repetitive genome of *Aegilops tauschii*, a progenitor of bread wheat, with the MaSuRCA mega-reads algorithm. *Genome Research*, 27:787–792. doi:10.1101/gr.213405.116
